# Reshoring silicon photovoltaics manufacturing contributes to decarbonization and climate change mitigation

**DOI:** 10.1038/s41467-023-36827-z

**Published:** 2023-03-08

**Authors:** Haoyue Liang, Fengqi You

**Affiliations:** 1 Systems Engineering, Cornell University, Ithaca, NY 14853 USA; 2 Robert Frederick Smith School of Chemical and Biomolecular Engineering, Cornell University, Ithaca, NY 14853 USA; 3 Cornell Atkinson Center for Sustainability, Cornell University, Ithaca, NY 14853 USA

**Keywords:** Climate-change impacts, Solar cells

## Abstract

The globalized supply chain for crystalline silicon (c-Si) photovoltaic (PV) panels is increasingly fragile, as the now-mundane freight crisis and other geopolitical risks threaten to postpone major PV projects. Here, we study and report the results of climate change implications of reshoring solar panel manufacturing as a robust and resilient strategy to reduce reliance on foreign PV panel supplies. We project that if the U.S. could fully bring c-Si PV panel manufacturing back home by 2035, the estimated greenhouse gas emissions and energy consumption would be 30% and 13% lower, respectively, than having relied on global imports in 2020, as solar power emerges as a major renewable energy source. If the reshored manufacturing target is achieved by 2050, the climate change and energy impacts would be further reduced by 33% and 17%, compared to the 2020 level. The reshored manufacturing demonstrates significant progress in domestic competitiveness and toward decarbonization goals, and the positive reductions in climate change impacts align with the climate target.

## Abstract (web summary)

Reshoring silicon photovoltaic manufacturing back to the U.S. improves domestic competitiveness, advances decarbonization goals, and contributes to mitigating climate change.

## Introduction

At the 2021 United Nations Climate Change Conference (COP 26) held in the U.K., attending countries agreed to sustain the goal of limiting global temperature rises to 1.5 degrees and to finalize the unfinished elements of the Paris Agreement^1–4^. The U.S. is ramping up its actions to create a carbon-free power sector by 2035 and to achieve “net-zero” greenhouse gas (GHG) emissions by 2050^5^. Facilitating this degree of climate change mitigation will require the accelerated deployment of fundamentally clean technologies, including sustainable solar photovoltaic (PV) technology, which marks the cleanest and cheapest form of electricity^6^. The growth of solar power has increased exponentially from small-scale applications to one of the mainstream sources of electricity, growing by more than 20% per year over the past six years^7,8^. The U.S. installed 14.9 GW_AC_ (19.2 GW_DC_) of PV in 2020, with PV accounting for approximately 40% of new electricity generation, up from just 4% in 2010^9,10^. By 2035, solar power is projected to support 40% of U.S. electricity demand, a tenfold increase over the solar output in 2021^11^. By 2050, solar could be nearly half of the electricity supply^11^. Solar power usage in the U.S. is also expected to reach 1067 TWh, 720% above the 130 TWh usage in 2020^8^. Following these projections, the market supply of PV technologies will be driven by energy policy goals and the aggressive pace of PV market demand.

The energy policy goals and the soaring PV panel demand impose a great supply challenge for the PV industry to catch up with the growing needs in the coming decades. Most U.S. PV installations relied on the use of imported panels, mainly coming from Asia^12^. Safeguard tariffs were placed onto imported silicon solar modules and applied for four years starting from 2018, with the duties declining 5% each subsequent year to arrive at 15% in the fourth year^6^, as part of a trend of trade protectionism regarding PV manufacturing. Around 96% of the world’s PV production comes from crystalline silicon (c-Si), which includes single-crystalline silicon (sc-Si), ribbon silicon (r-Si), and multi-crystalline silicon (mc-Si), representing the most widely used semiconducting materials^11,13,14^. While U.S. dependence on imported c-Si PV modules is non-negligible, the market favorability for PV imports is declining, as revealed by a decreasing fraction of imported PV panels despite a decrease in the tariff on imported silicon solar cells in recent years^11,12^.

Both supply instability and temporary trade restrictions due to geopolitical issues are the reasons behind the shift away of manufacturing from foreign supplies^15^. The effects of supply chain restructuring continue to propagate throughout the PV industry, as the overreliance on global trade structure emphasizes disruptions that jeopardize all countries involved^11,16^. While various countries locked their borders and international freighting took a nosedive in recent years due to the pandemic^17^, the demand for solar panels remains high due to elevated renewable electricity demand. Significant challenges have emerged in solar panel supply chains that face increasing risk from relying on external imports, which respond sensitively to any freight crisis or other potential disruptions^16^. Manufacturers want to move away from offshoring due to supply chain woes, geopolitical instability, freight cost, and many other considerations^18^.

The production of solar panels is expected to defer to more domestic sourcing, emphasizing self-sufficiency^11^, driven by growing geopolitical concerns and rapidly evolving trade relationships. Furthermore, reshored manufacturing facilitates close proximity to customers and markets, provides positive impacts on the domestic economy, and is also encouraged by policies and incentives^18^. The Inflation Reduction Act provides the necessary impetus for the domestic solar product manufacturing industry to catch up with countries that have outpaced the U.S. in clean energy technological development, innovation, and production^19^. The manufacturing tax incentives and investments, coupled with domestic content credits and the robust “Made in America” agenda, will provide the U.S. with the tools necessary to integrate climate and economic goals into multilateral trade discussions^20^. Past studies have focused on how reshored manufacturing could enhance solar PV product development, delivery performance, and cost leadership^21^, and the importance of the operation facilities in the PV panel reshoring decisions^22^. Despite the many existing studies related to reshored PV panel manufacturing decisions, the climate implications arising from the ongoing and upcoming solar panel value chain restructuring have not been systematically addressed with first-order analyses or otherwise been understood comprehensively. Whether reshored c-Si PV panel manufacturing, foremost a strategy to withstand supply disruptions, truly aligns with the ongoing climate target and energy policy to additionally accomplish climate change mitigation and energy consumption reduction remains a knowledge gap.

In this study, we perform the analysis to clarify the energy and environmental impacts of bringing c-Si PV production back to the U.S. by comparing the offshore (outsourced) manufacturing cases from 2010 to 2020 and the reshored (domestic) manufacturing scenarios from 2020 to 2050. To unilaterally quantify the impact of reshoring, we study the climate change impact of the hypothetical reshored scenario in 2020 and compare it to the offshore case in the same year. We also interpret the climate implications of a delayed reshoring schedule. To understand how the expansion of the solar panel industry that already greens the power grid could further promote climate change mitigation during its manufacturing stages together with the power of reshoring, we forecast reshored PV panel manufacturing scenarios with renewable penetration to the energy supply. The renewable penetration is also only made possible with the growing supply and demand of solar PV panels, as the ambitious outline would see solar electricity supply rising to 40% by 2035 before ultimately hitting 45% by 2050^11^. The prospective assessment of the decarbonization and climate change implications of reshored c-Si PV panel manufacturing in the U.S. to alleviate the supply chain woes emerging from unpredictable disruptions and geopolitical concerns is the major focus of this work. A comprehensive quantitative analysis of the detailed contribution of reshored manufacturing, renewable penetration as a result of solar PV industry growth, as well as other technological advancements in climate change mitigation and energy consumption reduction is provided. This work sheds light on some major policy implications. The implication of a positive reduction in climate impacts provides policymakers with a big picture conclusion that reshored manufacturing of PV panels aligns with the energy policy goals and contributes to the climate targets.Domestic c-Si PV panel manufacturing leads to a 23% reduction in climate change impact and a 4% decline in energy use from panel production compared to outsourced manufacturing because of reshoring. Bringing manufacturing back home helps the PV panel industry realize the decarbonization goal. If reshored PV manufacturing is achieved by 2035, the estimated GHG emissions and energy consumption from panel production would be 30% and 13% lower, respectively, than having relied on trading partners as in 2020. If the reshored manufacturing target is met by 2050, the climate impacts and energy use would then be reduced by 33% and 17%, as solar PV emerges as a major power source that characterizes the energy market for the remainder of the 21^st^ century. Domestic sourcing at a later date also aligns with the climate target and energy policy goals.

## Results and discussion

Reshored c-Si PV manufacturing tackles logistic challenges, but whether it directly reduces GHG emissions and energy use has not yet been discovered based on quantitative analysis. Exploring the climate change and energy impacts help us understand if reshored manufacturing aligns with the climate target. We perform a comparative and prospective life cycle assessment (LCA) study of several reshored manufacturing scenarios and outsourced manufacturing cases to examine the energy and climate impacts of fully eliminating dependence on foreign PV supplies. We define three cases (2010, 2015, 2020 offshore manufacturing) and seven scenarios (2020, 2025, 2030, 2035, 2040, 2045, 2050 reshored manufacturing). The reshored scenario in 2020 is studied to examine the climate impacts of solely bringing manufacturing back to the U.S. by comparing it with the outsourced manufacturing case in 2020. Moreover, reshored scenarios from 2025 to 2050 in 5-year increments are forecasted with cleaner power compositions such as wind, solar, geothermal, etc., building up from 21% renewable power contribution in 2020 to 42% in 2050^8^. We project and examine future scenarios spanning a wide range of time points from the near term to mid-century because of the potential uncertainties regarding the speed of domestic PV production scaling and the rate of equipment and workforce training expansion. Past studies expressed concern that trade restrictions and emphases on reshoring might slow the adoption of sustainable energy technologies, and the U.S. might not be fully equipped for rapidly upscaling domestic solar panel production^23,24^. Trade wars may also affect the environment by altering the global supply and consumption systems^25,26^, which become less conducive for less-developed regions to transition to clean energy^27^. Manufacturing efforts face an unpredictable future, and uncertainties remain regarding exactly when the reshoring of PV panels can be accomplished due to trade barriers, financing problems, workforce limitations, and so on. That is why multiple future reshored manufacturing scenarios at different time points, ranging from 2025 to 2050, are included in this study. These projections of different reshoring levels at various time points can be regarded as a sensitivity analysis to incorporate the temporal variations for when reshoring can be achieved. We study reshored manufacturing scenarios because legislations not only include targeted tax incentives aimed at manufacturing U.S.-sourced solar materials but also include key requirements around domestic sourcing. The Inflation Reduction Act opens up an opportunity for spurring U.S. solar technology supply chain as countries around the world race to lead the clean energy economy^28,29^. Reasonable predictions for these scenarios are made regarding the U.S.-centered domestic supplies as we foresee opportunities to grow a competitive supply chain of module components in regions like Alabama, Florida, Georgia, and so on^30^. We also study outsourced manufacturing cases in 2010 and 2015 to understand the impacts of the ever-changing global PV module supply chain structure on decarbonization.

For PV power plants, the majority of GHGs are emitted upstream of module manufacturing^31^. Solar panels do not produce emissions while generating electricity, but the operations and maintenance life cycle stage and the end-of-life treatment stage are included in this study to emphasize the relative emission reductions from panel manufacturing reshoring in the context of PV panel lifetime emissions. The operation and maintenance life cycle phase involves tasks like module cleaning, preventive maintenance (such as replacing inverters), as well as the repair of broken components, and the end-of-life treatment stage involves dismantling and shredding solar panels^32^. Based on the best available data^33,34^, a 1 m^2^ PV panel emits 0.27 kg CO2 eq GHG and demands 48 MJ of energy during its use stage, and emits 0.57 kg CO_2_ eq GHG and demands 74 MJ of energy at its end-of-life treatment stage. The energy and environmental impacts of the operations and maintenance life cycle stage and the end-of-life treatment stage can be useful to understand the relative emission reductions from PV panel manufacturing in the overall context of PV panel life cycle.

### Reshoring as a decarbonization strategy

**Fig. 1:**
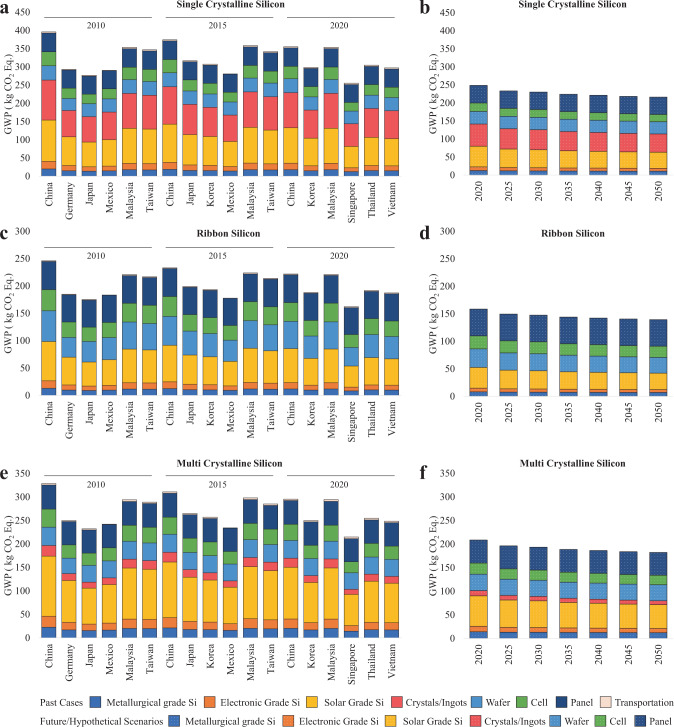
Greenhouse gas emissions of crystalline silicon photovoltaic (PV) panel supplies to the U.S., with the breakdown of climate change impacts of each individual manufacturing stage and transportation. Results are presented for (**a**) single-crystalline silicon (sc-Si) PV in offshore cases, (**b**) sc-Si PV in reshored scenarios, (**c**) ribbon silicon (r-Si) in offshore cases, (**d**) r-Si in reshored scenarios, (**e**) multi-crystalline silicon (mc-Si) in offshore cases, as well as (**f**) mc-Si in reshored scenarios. Reshored manufacturing scenarios in (**b**, **d**, and **f**) illustrate the downward climate change impact trend over time, whereas offshore manufacturing cases in (**a**, **c**, and **e**) do not guarantee climate change mitigation over time, as illustrated by the higher emissions in 2015 than in 2010 in some regions. To study the impact of reshoring in 2020, we compare the 2020 case and the 2020 scenario. The sources of PV supplies in the 2020 case include China, South Korea, Malaysia, Singapore, Thailand, and Vietnam, as shown in (**a**, **c**, and **e**). The PV panels in the 2020 scenario are only manufactured in the U.S., as shown in (**b**, **d**, and **f**). We see a reduction of 23% global warming potential from PV panel manufacturing on average as a result of reshoring.

In this study, climate change mitigation potential and energy performance of PV panel manufacturing are presented to study the energy and decarbonization impacts of reshoring on solar panel production. The quantitative analysis is conducted based on two important climate-related metrics, global warming potential (GWP) and cumulative energy demand (CED)^35^. To investigate the impact of switching from offshore manufacturing to domestic production on the c-Si PV panels, we compare the reshored scenario (Fig. 1b, d, f) in 2020 with the outsourced case (Fig. 1a, c, e) in the same year. Figure 1a, c, and e present the GHG emissions for production across the entire supply chain for each trading partner (China, Malaysia, Japan, etc.) from silica sand production to panel manufacturing between 2010 and 2020 for three types of c-Si materials, and emissions from shipping of these panels to the U.S. are also included. Figure 1b, d, and f showcase the emissions from U.S. domestic production of PV panels from 2020 to 2050 in five-year increments. Compared with relying on global supplies (offshore case) in 2020, domestic manufacturing of c-Si PV modules in the U.S. reduces GHG emissions by 23% and energy use by 4%. The offshore case in 2020 mainly relied on supplies from Malaysia (38%), Vietnam (21%), Thailand (17%), South Korea (9%), China (6%), and Singapore (3%)^36,37^. Manufacturing PV panels in Malaysia under the 2020 offshore case generates 42% more GHG emissions than manufacturing in the U.S., mainly due to the high emissions (26%-29% of all emissions) from solar grade silicon manufacturing stage for all three types of c-Si technologies (Fig. 1a, c, e), and the sc-Si crystal production stage that generates 26% to 28% of all emissions for sc-Si technology (Fig. 1a).

**Fig. 2:**
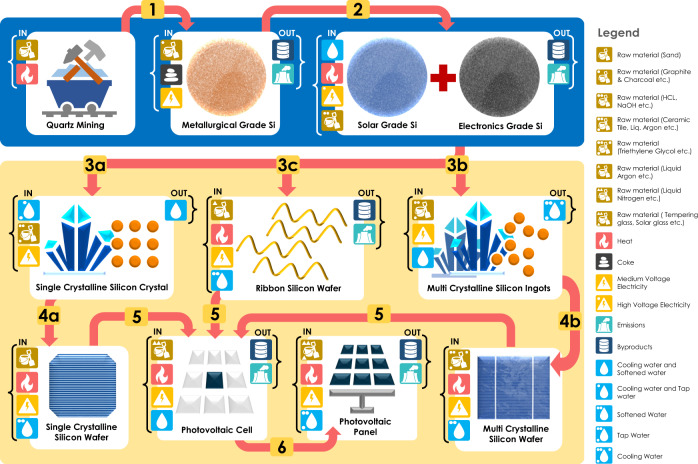
Crystalline silicon photovoltaic panel manufacturing stages. The output from quartz mining stage is the silica sand, which is the input for the metallurgical grade silicon production (step 1). Metallurgical grade silicon is the input of both solar grade silicon production and electronics grade silicon production (step 2). Three types of crystalline silicon materials go through different paths for wafer production (step 3a & 4a for single-crystalline silicon, step 3b & 4b for multi-crystalline silicon, and step 3c for ribbon silicon). Photovoltaic cells are made from wafers (step 5), and photovoltaic panels are made from cells (step 6).

Although the Malaysian government launched its Green Technology Policy in July 2009 to encourage and promote the use of renewable energy for Malaysia’s sustainable development^38^, almost half of its power generation still relied on coal (46%) a decade later after the policy was launched^39^. On the other hand, the U.S. relied heavily on natural gas (39%), which contributes almost twice as much to total electricity generation as coal source (20%)^39^. Besides the quartz mining stage, all stages require the use of electricity, as shown in Fig. 2. Among them, more high-voltage electricity power is needed in solar grade silicon manufacturing stage, which is on average six to ten times the amounts needed for electronics grade silicon and metallurgical grade silicon production. The power sector is one of the major sources of GHG emissions. The differences in power mixes between countries lead to discrepancies in climate change impacts of silicon manufacturing, which directly results in the gap in GHG emissions between the outsourced case and domestic scenario in 2020.

**Fig. 3:**
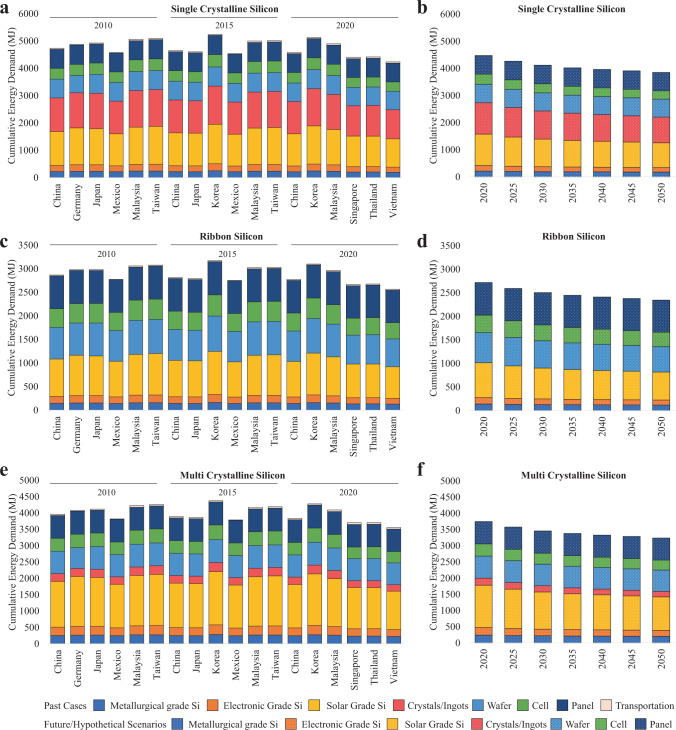
Cumulative energy demand of crystalline silicon photovoltaic (PV) panel supplies to the U.S., with the breakdown of climate change impacts of each individual manufacturing stage and transportation. Results are presented for (**a**) single-crystalline silicon (sc-Si) PV in offshore cases, (**b**) sc-Si PV in reshored scenarios, (**c**) ribbon silicon (r-Si) in offshore cases, (**d**) r-Si in reshored scenarios, (**e**) multi-crystalline silicon (mc-Si) in offshore cases, as well as (**f**) mc-Si in reshored scenarios. Reshored manufacturing scenarios in (**b**, **d**, and **f**) illustrate the downward energy consumption trend over time, whereas offshore manufacturing cases in (**a**, **c**, and **e**) show that energy consumption can be higher when supplies change, as illustrated by the higher energy use in 2015 of some suppliers than in 2010. To study the impact of reshoring in 2020, we compare the 2020 case and the 2020 scenario. The sources of PV supplies in the 2020 case include China, South Korea, Malaysia, Singapore, Thailand, and Vietnam, as shown in (**a**, **c**, and **e**). The PV panels in the 2020 scenario are only manufactured in the U.S., as shown in (**b**, **d**, and **f**). We see a reduction of 4% energy use from PV panel manufacturing on average as a result of reshoring.

Similarly, the energy performance of offshore cases (Fig. 3a, c, e) and reshored scenarios (Fig. 3b, d, f) are presented. Figure 3a, c, and e, similar with Fig. 1a, c, and e, exhibit the sources of PV supplies in cases from 2010 to 2020 across the entire supply chain for each trading partner (China, Malaysia, Japan, etc.), while Fig. 3b, d, and f showcase the scenarios in which production occurs in the U.S. itself. We see a 4% reduction in energy consumption when switching from offshore to reshored manufacturing in the same year, despite the decline in energy use being less significant than that of climate change mitigation. As opposed to the climate change impacts of reshored manufacturing in the U.S., which always results in lower GHG emissions than all offshore suppliers (30% lower than China, 17% lower than South Korea, 3% lower than Singapore, 18% lower than Thailand, as shown in Fig. 1), the energy consumption in the U.S. in the reshored scenario is not always lower than all suppliers in the offshore case in 2020, as shown in Fig. 3. Specifically, manufacturing c-Si PV in the U.S. requires more energy use than some of the suppliers in the outsourced case, such as Singapore (2% lower than the U.S.), Thailand (1% lower than the U.S.), and Vietnam (5% lower than the U.S.) (Fig. 3).

We explore the reasons behind the lower energy footprint of manufacturing PV panels in offshore cases. Vietnam relied heavily on hydropower (28%), which is a much more efficient way to generate electricity than coal and natural gas. Modern hydro turbines can convert as much as 90% of energy into electricity, whereas the best fossil fuel plants are only 50% efficient^40^. Thus, less energy is required to manufacture PV panels in Vietnam due to more efficient energy usage. Meanwhile, transportation accounts for just 1% of CED, a minimal amount compared to the manufacturing stages. After oceanic shipping (1% of CED) is included for supplies from Vietnam, the total energy usage is still 5% lower than reshored manufacturing in the U.S. Despite the similar energy performance of production in Thailand and Singapore which also relied heavily on natural gas, as well as the lower energy use of c-Si PV production in Vietnam, the offshore manufacturing case still results in 4% higher CED compared with the reshored scenario in the same year. As mentioned earlier, the U.S. only attributed 41% of its PV supplies from these three countries in 2020 (21% from Vietnam, 17% from Thailand, and 3% from Singapore);^36^ the weighted average energy use when the U.S. relied on foreign suppliers is still higher than domestic production. However, reshored manufacturing does not guarantee an absolute energy performance advantage compared to offshore manufacturing due to the proximity (4% variation) of energy consumptions under the reshored scenario and the weighted average outsourced case.

### Future reshored manufacturing scenarios with renewable penetration to the power grid

Meeting the increasing demand for green power worldwide, growing shares of renewable energy sources over time as well as switching from global sourcing to reshored manufacturing would lead to greater climate change mitigation from c-Si PV module production in the future. The growing shares of renewable energy sources will not be possible without the increasing demand and supply of c-Si PV panels. Renewable penetration and expansion of c-Si PV panel manufacturing facilitate each other to achieve climate benefits. The amount of GHG emissions generated from reshored c-Si PV module production in the U.S. in 2050 is anticipated to reduce by 33% compared to relying on foreign supply in 2020 and 30% lower in 2035 than in 2020 (Fig. 1). The forecasted significant climate change mitigation is fulfilled by both reshoring manufacturing back to the U.S. and the large renewable penetration to the power grid, which is anticipated to happen in the U.S. in the next few decades^41^. The usage of renewable energy, including wind, solar, geothermal, etc., contributes to a 470% to 520% greater fraction of energy in 2050 than in 2010, exemplifying the far-reaching impacts of penetration of renewables into the power mix on CED impact analysis^8^. Compared to 2020, the coal-sourced share of electricity generation in the U.S. is projected to decrease by 18% in 2030, 33% in 2040, and 43% in 2050, while the nuclear source share would decrease by 27% in 2030, 37% in 2040, and 44% in 2050^8^, as shown in Supplementary Methods 2: Electricity mix. As the U.S. transitions to greener sources of electricity, it is projected to rely on wind nearly twice as much starting merely from 2024, compared to the 2020 level. Among the renewable fuels, solar power is anticipated to increase by eightfold from 132 billion kWh in 2020 to 1071 billion kWh forecasted in 2050^8^ (Supplementary Fig. 11). GHG emissions decrease appreciably over time as a result of both reshoring and the progression to more renewable power generation sources as a result of reshoring.

**Fig. 4:**
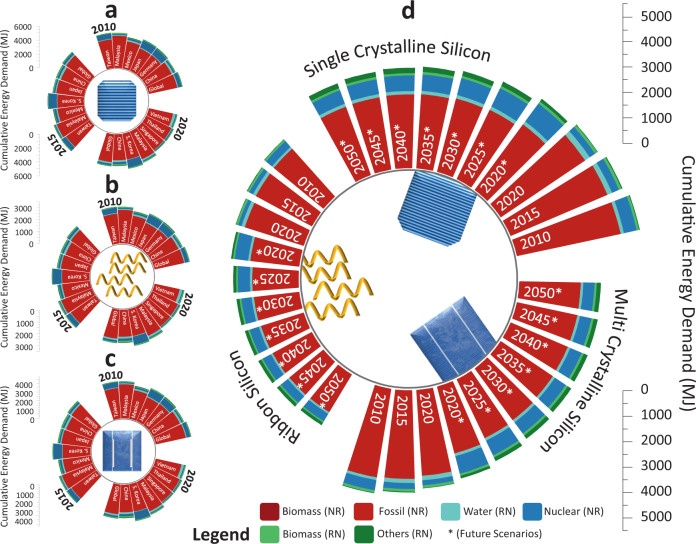
Impact analyses of the energy consumption of photovoltaic panel supplies to the U.S. Three cases in 2010, 2015, and 2020 are presented based on three types of crystalline silicon photovoltaic technologies: (**a**) single-crystalline silicon, (**b**) ribbon silicon, (**c**) multi-crystalline silicon. **a**–**c** represent the energy use for production across the entire supply chain for each trading partner. Seven scenarios in 2020, 2025, 2030, 2035, 2040, 2045, and 2050 are presented altogether in (**d**), with * indicating future scenarios.

Despite the climate change mitigation, our results also shed light on the significant energy performance improvements. Compared with relying on global supplies in 2020, we project that domestic manufacturing of c-Si PV modules in the U.S. in 2035 and 2050 requires 13% and 17% less CED (Fig. 3 and Fig. 4), including 32% less non-renewable fossil energy (Fig. 4), indicating a significant energy reduction trend resulting from supply transition. Based on the projections on the energy decarbonization transition that happens alongside reshoring, we see not only larger shares of renewables accounting for primary energy consumption but, resultantly, overall lower primary energy consumptions over the years for all c-Si technology, as shown in Fig. 4.

### Past offshore manufacturing cases

**Fig. 5:**
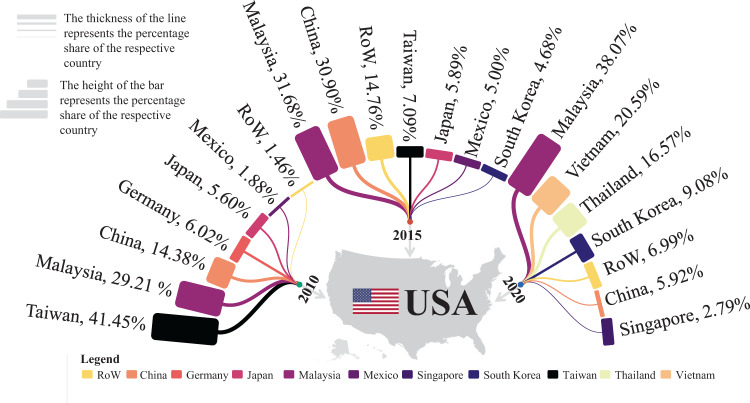
Supply chain structure of solar photovoltaic panels shipped to the U.S. in 2010, 2015, and 2020. The suppliers involved in the 2010, 2015, and 2020 outsourced cases are determined based on the market share data. The U.S. mainly imported solar photovoltaic products from Asian trading partners. The market shares of module supplies changed dramatically over the years due to geopolitical tensions, safeguard tariffs, policies, etc. The rest of the world (RoW) includes all other suppliers other than the top six suppliers.

The trade structure significantly changed over the past few years, leading to an increased GHG emission from 2010 to 2015 (Fig. 1). The U.S. mainly relied on PV supplies from Taiwan (41%), Malaysia (29%), China mainland (14%), Germany (6%), Japan (6%), and Mexico (2%) in 2010, and Malaysia (32%), China mainland (31%), Taiwan (7%), Japan (6%), and Mexico (5%) in 2015, as shown in Fig. 5. The supply share of the PV system in Taiwan drastically reduced from 41% to 7%, and that of China mainland went up from 14% to 31% from 2010 to 2015, while other regions’ shares changed over time but not to any extent that would drive significant impacts (Fig. 5).

The GHG emissions from panel production of the 2015 outsourced case are estimated to be even higher than the 2010 outsourced case. Although the life cycle carbon footprint of PV systems in China decreases by 5% (20 kg CO_2_ equivalent per 1 m^2^ of PV module manufactured) in 2015 compared to 2010, the U.S. imported more panels from mainland China in 2015 (31%) than in 2010 (14%). The GHG emissions from PV panel production in mainland China in 2015 are lower than in mainland China in 2010, but are still higher than the other suppliers (Germany, Japan, Mexico, Malaysia, Taiwan) in 2010, as shown in Fig. 1a, c, e, leading to a higher weighted sum of emissions from trading partners in 2015 than in 2010. Therefore, offshore manufacturing does not guarantee decreasing GHG emissions over time. The fluctuating GWP over the years is highly relevant to the displacements in the importing supply share of c-Si PVs. A supply chain crisis can occur at anytime that threatens the growth of the solar energy and the PV industry or even leads to increased GHG emissions in c-Si PV manufacturing. To stabilize the supply and to attain consistent emission reduction, reshoring is an option to consider, and growing efforts of reshoring manufacturing have been demonstrated^5^.

Similarly, the energy consumption of manufacturing PV panels in 2015 in some countries is higher than in the 2010 outsourced case. For instance, South Korea was not a major source of PV supplies to the U.S. in 2010, but it became one of the top six suppliers in 2015. Manufacturing PV panels in South Korea in 2015 requires 5% more energy than the average 2010 case, while Malaysia and Vietnam in 2015 requires 2% to 10% more energy than most suppliers (China, Germany, Japan, Mexico) in 2010. This indicates that when manufacturing locations shift from China, Germany, Japan, and Mexico in 2010 to South Korea, Malaysia, and Vietnam in 2015, the energy usage increases, as shown in Fig. 3. Clearly, with the ever-evolving pace of imported freight, the future of worldwide module production and the PV supply chain is uncertain, just as it was so easily disrupted by the supply crisis due to the COVID-19 pandemic^42^. Since the U.S. economy has faced many supply bottlenecks that contribute to high inflation, a strategy that ensures domestic manufacturing in the U.S. is encouraged^43^. As the U.S. PV demand growth continues, there might be an opportunity for further domestic manufacturing expansion, particularly given the potential supply chain disruption^11^.

### Contributors to the climate of reshoring PV manufacturing

**Fig. 6:**
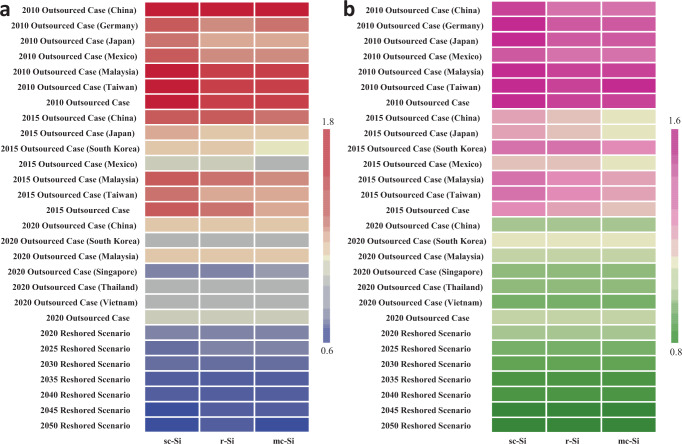
Carbon emission factor/greenhouse gas emission factor and energy payback time of outsourced cases (2010, 2015, 2020) and reshored scenarios (2020, 2025, 2030, 2035, 2040, 2045, 2050). The switch to reshored manufacturing and increasing renewable penetration to the power grid work together to drop (**a**) carbon emission factor and lower (**b**) energy payback time (EPBT) of manufacturing c-Si PV panels. The presented results are normalized and vary based on the colors shown on the color bar. In particular, the red and pink colors represent high carbon emission factors and high EPBT, suggesting more energy and environmental burdens, while the blue and green colors represent low carbon emission factors and low EPBT, indicating lower burdens. The 2020 outsourced case is defined as the reference for normalization.

Together with the impacts from reshoring and renewable penetration, we incorporate other factors with temporal or geographical variations, such as module efficiency, performance ratio, solar irradiation, and grid efficiency, in our analysis to study the energy and environmental impacts of these factors considered. Based on the **Parameters** under “**Methods**”, we estimate the carbon emission factor and the energy payback time (EPBT) of outsourced cases and domestic scenarios. We see that while other factors are taken into consideration, as illustrated in Fig. 6, the estimations of these two metrics also differ drastically between cases that rely on foreign supply and scenarios that depend on domestic production. As it stands, reshoring PV panel manufacturing sees a drastic reduction of carbon emission factor of 31% in 2035 and 33% in 2050 and EPBT decline of 14% in 2035 and 17% in 2050, compared with the 2020 offshore case (Fig. 6). The reductions can chiefly be accredited to the switch to reshored manufacturing and the changing breakdown of energy sources in the U.S. If reshored manufacturing can be achieved in 2035, among 31% of the reduction in carbon emission factor, reshoring leads to 23% of emission factor decrease while renewable penetration to the power grid contributes to 8%. On the other hand, when it comes to reductions in EPBT, 4% of EPBT decline is attributed to reshoring, while the remaining 10% are the credits of renewable penetration. We see that the act of reshoring has a greater impact on carbon emission reduction. The renewable penetration anticipated in the U.S. has a more significant influence on EPBT, but the projections on renewable penetration, including the soaring solar energy, can only be implemented if the PV panel supply surge in the next few years. Therefore, renewable penetration and reshored manufacturing for increasing PV demand are not mutually exclusive but rather in the same boat. They work together to drop carbon emission factors and lower EPBT of manufacturing c-Si PV panels.

### Sensitivity analysis

**Fig. 7:**
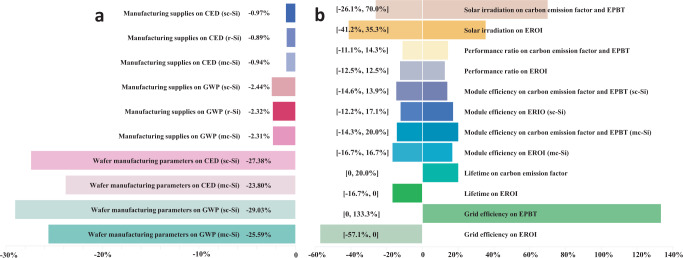
Sensitivity analysis of various parameters on cumulative energy demand, global warming potential, carbon emission factor, energy payback time, and energy return on investment estimations. The sensitivity analysis includes: (**a**) manufacturing supply chain structure (number of representative trading partners) and wafer manufacturing parameters on cumulative energy demand (CED) and global warming potential (GWP), (**b**) solar irradiation, performance ratio, module efficiency, lifetime, and grid efficiency on energy payback time (EPBT), carbon emission factor, and energy return on investment (EROI).

Besides our main analysis, we also study how other factors can have an impact on the energy and climate change profiles. We perform sensitivity analysis of various parameters on CED, GWP, carbon emission factor, EPBT, and energy return on energy invested (EROI) estimations. We first explore whether choosing the top six suppliers to represent the trading partners would or would not lead to significant bias in the energy and climate change results. As an additional litmus test for the number of global PV manufacturing locations to represent offshore locations, to ensure that the six suppliers chosen to represent the global supply sourcing market are appropriate, we conducted a sensitivity analysis on the number of suppliers designated in the market share calculations. We perform a sensitivity analysis specifically for the 2015 offshore case since the global supply of c-Si PV to the U.S. is less distinctly dominated by the top six suppliers (85% of supplies come from the top six) in 2015, whereas it was well-dominated by the top six in 2010 (99% from top six) and 2020 (93% from top six), as shown in Fig. 5. To perform this sensitivity analysis that includes more spatial variation, we study and compare the energy and environmental impacts of the top nine suppliers. Alongside the top six locations, we include Germany (4.07%), Singapore (3.16%), and Vietnam (2.90%) as the top suppliers. The calculated energy profile represented by CED decreases by less than 1% (0.97% for sc-Si, 0.89% for r-Si, and 0.94% for mc-Si), while environmental impact represented by GWP decreases by less than 2.5% (2.44% for sc-Si, 2.32% for r-Si, and 2.31% for mc-Si), as shown in Fig. 7a. The results thereby affirm that designating just the top six exporters as the major market share components is a succinct yet sufficiently representative group by which to assess the c-Si PV supply chain and trade structure before 2020.

We also perform sensitivity analysis of key parameters involved in the wafer manufacturing stage on energy and climate change impact. As an example of such an analysis, we show how reduced wafer thickness and kerf losses for slurry-based sawing would create an impact on CED and GWP when the wafers were manufactured in China in 2020. We find that the decline of these two parameters leads to a lessened amount of casted silicon and Czochralski silicon ingots needed for wafer production, which further leads to decreasing wafer weight input for PV cell and module production^44^. As a result, such changes in wafer thickness and kerf loss would cut CED by 27% (sc-Si) and 24% (mc-Si) and GWP by 29% (sc-Si) and 26% (mc-Si), as shown in Fig. 7a. On the other hand, according to the PV report by the Fraunhofer Institute for Solar Energy Systems that summarized the wafer thickness of c-Si PV cell development over the years^45^, the wafer thickness changed by no more than 5% from 2015 to 2020. Thus, we aim to mainly study and emphasize energy structure transitions and PV supplies instead, and the analysis regarding wafer thickness and kerf losses are considered in this sensitivity analysis.

We further investigate how variations in solar irradiation, performance ratio, module efficiency, lifetime, and grid efficiency can influence EPBT, carbon emission factor, and EROI. If grid efficiency increases from 30 to 70%^46,47^, EPBT can increase by 133%, and EROI can decrease by 57%, as shown in Fig. 7b. We see that through technological advancements, module efficiency is also anticipated to go up as a result of panel design improvements^6^, and we see an up to 17% increase in EROI and up to 15% decrease in carbon emission factor and EPBT as a result of improved module efficiency. We also take into consideration the degradation rates of c-Si PV panels which are reflected in their decreasing module efficiencies^48^. The results show that panel degradation leads to a 12% reduction in EROI for sc-Si and a 17% decrease for mc-Si, as well as a 14% increase in carbon emission factor and EPBT for sc-Si and a 20% increase for mc-Si. Another technological advancement we consider is the performance ratio^49^, which can lead to an 11% decrease in carbon emission factor if it reaches 90% and a 14% increase if it drops to 70%. Furthermore, we study the impacts of geographical location on these metrics from various levels of solar irradiation^47^. Regions with low solar irradiation come with higher carbon emission factors and EPBT, as shown by the 70% increase compared to medium irradiation regions, while regions with high solar irradiation demonstrate 26% lower carbon emission factor and EBPT. If the lifetime of the c-Si PV panel decreases by five years, the carbon emission factor would thus go up by 20%. The sensitivity analysis of various parameters can help us understand how technological advancements and geographical locations that place alongside manufacturing in the U.S. can have an impact on the energy and climate change metrics.

Reshoring c-Si PV manufacturing plays an important role in mitigating climate change. This study investigates the long-term implications of the current trend toward building a resilient and reliable reshored PV manufacturing supply. Departing from foreign supplies and instead bringing manufacturing back home will provide the PV industry with an alternative to fall back on when disruptions resurface. Reshored PV panel manufacturing is not only a strategy to protect domestic industry from supply bottlenecks but also aligns with the ambitious climate policy by substantially reducing carbon emissions.

### Insights

In this study, the offshore manufacturing cases from 2010 to 2020 are considered as the U.S. previously relied on major PV panel imports from Asia^12^. The reshored manufacturing scenario in 2020 is studied unilaterally to examine whether the “Make it in America” strategy alone can support the climate agenda to realize decarbonization goals when compared with the offshore manufacturing case in the same year. More reshored manufacturing scenarios in 2025, 2030, 2035, 2040, 2045, and 2050 are forecasted to study how the reshored strategy and the climate policy interact in the next few centuries, given the disruptive nature that global politics could have on the PV supply chain.

Reshoring manufacturing reduces climate change impact from PV panel production by 23%, leading to tremendous benefits for the climate. Manufacturing and trade policies, significant financial support and incentives, as well as strategic actions focused on the workforce, will facilitate the rebuilding and continued operation of PV panel manufacturing facilities^11^. As the U.S. PV demand growth continues in the future, there may be opportunities for future domestic manufacturing expansion. If the reshored manufacturing can be achieved in 2035, a 30% climate change mitigation from manufacturing c-Si PV panels is expected. If the reshored manufacturing can be realized in 2050, a 33% mitigation of the climate change impact from panel production is projected. These reductions matter not only in the manufacturing stage but also in the overall scheme of the PV panel life cycle. The manufacturing stage of the c-Si PV life cycle is where the majority of GHG emissions occur^31^. Compared to the manufacturing stage, the operations life cycle stage of the solar PV system generates modest GHG emissions that are close to zero, due to the relatively low operational and maintenance requirements of PV systems^31^. A 1 m^2^ PV panel emits 0.27 kg CO_2_ eq GHG and demands 48 MJ of energy at its use stage^33,34^. Similarly, the amount of GHG emissions generated from the end-of-life treatment stage is minimal. A 1 m^2^ PV panel emits 0.57 kg CO_2_ eq GHG and demands 74 MJ of energy at its end-of-life treatment stage^33,34^. Putting them in the overall context of PV panel lifetime emissions and energy impact, the GHG emissions from the use stage contribute to less than 0.20% of PV lifetime emissions, and those from the end-of-life management stage contribute to less than 0.41%, as shown in Supplementary Fig. 4 under Supplementary Discussion 1: Manufacturing vs operations and maintenance vs end-of-life treatment. The energy use from the use stage contributes to less than 2.0% of lifetime energy use, and that from the end-of-life management stage contributes to less than 3.0%. Since most carbon emissions occur in the upstream manufacturing process, and contributions of emissions from the use stage and the disposal stage are generally low, we conclude that the climate change mitigations from panel manufacturing as a result of reshoring are significant in the overall lifetime emissions.

Based on our quantitative analysis, reshoring aligns with the ambitious climate target. As solar is expected to make up 40% of U.S. power by 2035 and up to 70–80% by 2050, this can only be made possible by producing more PV panels^11^. Although there are various emerging PV technologies, no alternative PV technology can displace c-Si quickly enough to achieve power sector decarbonization by 2035^50^. Developing the U.S. c-Si PV domestic supply could mitigate challenges related to production disruption, compete with demand from other industries or countries, and maintain a robust U.S. domestic solar manufacturing leadership^51,52^. The Inflation Reduction Act encourages U.S. supply chains to span clean technologies, including solar technology, to create opportunities for small businesses and invest in American workers and the PV industry^53^. The legislation offers specific tax incentives for businesses that manufacture solar products domestically, and includes important requirements around domestic sourcing, such as the use of domestic panels in solar projects, as well as around prevailing wages and apprenticeships to ensure that good-paying jobs are offered to boost American manufacturing and competitiveness^20^. Policymakers have also stepped-up attempts to restart the American PV industry to renew efforts to bring manufacturing back. Such proposals draw on the momentum of a growing domestic movement in support of a “Green New Deal”, which has promised decent manufacturing jobs as a result of investments in low-carbon technologies and increasingly justified climate policies^54^. As of now, some policies explicitly aim at reshoring, such as tariffs put in place by past administrations, are still in effect, and a broad investigation into gaps in domestic supply chains has also been launched^54^.

Offshore manufacturing in the past does not always align with the climate target. Apart from the reshored manufacturing scenarios in the future that are assumption driven and formulated based on reasonable predictions, we also examine the past outsourced cases that relied on global supplies to interpret the impacts behind the ever-changing supply chain and manufacturing locations, as well as the power mix of trading partners from 2010 to 2020. Manufacturing c-Si PV panels from outsourced locations result in more GHG emissions in 2015 than in 2010. Moreover, as manufacturing locations shift from China, Germany, Japan, and Mexico in 2010 to South Korea, Malaysia, and Vietnam in 2015, the energy usage from panel production increases by up to 10% as well. As global dynamics shift quickly and more and more emerging supply crises demand our attention, it is difficult to determine an outsourced procurement strategy that not only complies with the ambitious national climate policies but also assures that geopolitical tensions would have no influence on it. Supply disruptions and bottlenecks can occur at any time to threaten the growth of renewable solar power and the PV industry, and the changing manufacturing deployment on account of policies and demand can increase GHG emissions. To stabilize supply and attain consistent carbon emission reduction, switching to leading-edge domestic manufacturing is an option to consider. Doing so will enable the pursuit of strategic objectives, particularly those in the energy, climate, and national security domains.

Manufacturing c-Si PV panels is attractive to pursue domestically as reshored production demonstrates many more benefits. The domestic production of solar products also aids in building broader coalitions and offers possible spillover benefits for climate policy. Outsourcing production to other countries over time is not a sustainable business model^55^. Offshoring can potentially result in job losses, wage reduction, and disruption of business innovation and productivity, which leads to policymakers proposing anti-offshoring or reshoring bills and policies that provide tax incentives for domestic production^56^. A reshored manufacturing base in solar PV may provide benefits such as more direct local employment and a more resilient energy supply system. Foreign manufacturers may be risky, impractical, or undesirable partners for public funds, whereas establishing a strong link between public funding of research and development and the domestic private sector has been identified as crucial to achieving climate goals, both by lowering the risks of scale-up and by granting access to markets^57^. The reshoring decision and climate agendas harmonize to ramp up climate actions, as carbon emission factors and EPBT of c-Si PV reshored manufacturing in the future reduce dramatically. If reshored manufacturing can be achieved in 2035, among 31% of the reduction in carbon emission factor from panel production, reshoring leads to a 23% of emission factor decrease while renewable penetration to the power grid contributes to 8%. On the other hand, among 14% of EPBT reductions from panel manufacturing, 4% of EPBT declines are attributed to reshoring, while the remaining 10% are the credits of renewable penetration. Renewable penetration and reshoring work together to create enormous energy and climate benefits. Renewable penetration to the power grid can only be made possible through more c-Si PV solar panel manufacturing and can only be achieved as the solar panel industry expands. As the U.S. achieves its energy transition goal by 2035 and 2050, the reshored panel manufacturing will benefit from large shares of renewables in the power grid by then in return. Besides energy and environmental strengths, the import costs of c-Si manufacturing inputs add 11% to the total expenditure, while a build-up in the domestic PV supply chain from “cradle-to-site” would dramatically reduce the cost^11^. Despite the minimized cost, c-Si PV manufacturing materials are mostly benign and available in very large quantities and have demonstrated long-term durability^11^. Besides, along with reshored manufacturing and increasing renewable power sources, technological innovations and breakthroughs can help achieve lower carbon emission factors and EPBT (15% by module efficiency advancement, 11% by performance ratio improvement) from PV panel production by 2050. Apart from withstanding supply crisis, reshored manufacturing is appealing to implement due to the numerous advantages listed above, which can be harmonized with technological advancements and renewable penetration to the power grid, and the conclusion of this study has important implications for other regions or industries to secure a reliable supply base.

## Methods

### Methodology overview

In our LCA to study c-Si PVs, we follow the existing approach of setting the functional unit as a 1 m^2^ c-Si PV module over the “cradle-to-site” PV life cycle system boundary from quartz mining to PV module production to shipment of PV panels^58^. The study is conducted in the OpenLCA 1.10.3 software with data imported from ecoinvent version 3.7.1 (2020) database and OpenLCA LCIA methods version 2.0.5^34^. We also used Microsoft Excel version 2212 for data analysis. The LCA we conducted is spatially explicit with all geographically specific inventories, mainly including raw materials and energy. We discuss the various elements of our approach: (1) elements of LCA, including goal and scope definitions, life cycle stages, life cycle inventory, and life cycle impact assessment; (2) integration of the global industrial structure change; (3) energy structure and compositions of power mix; (4) parameters for EPBT and carbon emission factor calculations. In this study, we focus on PV modules ultimately shipped to or manufactured in the U.S. We investigate all scenarios and cases applying three types of c-Si based technologies toward 2050, namely sc-Si, r-Si, and mc-Si, as shown in Supplementary Fig. 8 under Supplementary Methods 1: Life cycle inventory and life cycle stages.

### System boundary and analysis scope

In this work, we aim to evaluate the “cradle-to-site” climate change and energy impacts of reshored c-Si PV panel manufacturing to assess if the act of bringing manufacturing back home aligns with the climate target. The system boundary of the life cycle of c-Si PVs consists of several stages, from raw material acquisition to solar module production. We include all major and minor manufacturing and construction materials, from wet wood chips in quartz mining to low-iron solar glass in module production, in the inventory. To enable a fair comparison of modules in different cases and scenarios regarding material inputs, energy consumption, and emissions, we define the system boundary to be from silica sand mining to panel manufacturing to shipping panels to the U.S. for all for consistency. In this study, the overarching functional unit, typically defined in terms of a unit quantity of product, is set to be 1 m^2^ of the solar module according to the previous literature^47,59^, which is helpful to capture the changes in energy and environmental profiles proportional to PV size directly over time. We note that the end-of-life phase is excluded from the system boundary following assumptions in existing literature accounting for lack of data^7^, as there has been insufficient data on the disposal phase as well as the balance of plants^60^.

### Life cycle stages

The manufacture of PV modules involves several stages, from quartz mining to PV module production, as shown in Fig. 2. The system starts with silica sand acquisition, of which only heat and sand are added to the first stage to obtain silica sand^61^. Metallurgical grade silicon, a crucial stepping stone in the refining process of silicon metals, is then yielded by a carbothermic reduction reaction from silica sand with other material inputted, including petroleum coke, wet wood chips, etc., into the second stage^62^. After metallurgical grade silicon is obtained, electronics grade silicon is produced through the Siemens process, which involves the deposition of silicon from a mixture of purified silane with an excess of liquid hydrogen onto high purity metallurgical grade silicon. Solar grade silicon is produced through a modified Siemens process, which involves additional processing to separate the toxic and corrosive gas from the reduction process of metallurgical grade silicon^63,64^. These procedures to obtain all these types of silicons are homogeneous regardless of c-Si technology type, although the quantities needed to produce the same functional unit of three types of c-Si PV modules are different. After solar grade and electronics grade silicon are obtained, the manufacturing configurations of PV systems start to differ by the type of c-Si selected as the semiconductor material to form cells and modules. When sc-Si is the semiconductor material, the Czochralski crystal growth technique is implemented to form sc-Si crystal blocks in an inert atmosphere, such as argon in this study^65^. These crystals then go through the wafer sawing process in that individual silicon chips are mechanically separated from each other for cell manufacturing^66^. When r-Si is the semiconductor material, solar grade silicon and electronics grade silicon are used directly for r-Si wafer production, of which carbon-based strings are pulled upward through holes with molten silicon, and sawing loss is avoided^67^, leading to relatively low energy required to manufacture r-Si PV module compared with sc-Si and mc-Si technologies. When mc-Si is picked as the semiconductor material, solar grade silicon and electronics grade silicon are melted and cast into quartz crucibles to form mc-Si ingots^68^. Similar to sc-Si crystals, mc-Si ingots then go through the process of wafer sawing^69^. Processing of silicon wafers into solar cells involves texturing, acid cleaning, diffusion, etching, etc., while electrical contacts are placed between the cells and then wired and arrayed to form modules. Despite the differences in wafer types, the cell manufacturing and module assembly processes are similar for all three types of c-Si technologies^70^.

### Life cycle inventory

The LCI in our study embraces mass and energy balance involved in each life cycle stage, integrating the trade structure of products as well as power mix inputs. We derive supply chain structure from the trade data for c-Si PV cells and general solar cells assembled into modules or panels^36^, and more details are discussed under Supply chain structure and information. The relevant LCI regarding energy information of global PV exporting regions in the past, as well as power mix predictions in the future are obtained, which are discussed in more detail under Energy structure^8,39^. General mass and energy balance data throughout the life cycle of PV are collected from ecoinvent version 3.7.1 and relevant literature^34^.

### Life cycle impact assessment

Primary energy consumption and the intergovernmental panel on climate change (IPCC) 2013 method are selected as the primary LCIA methods in this study, as it is imperative to comprehend the energy and decarbonization implications of promising energy suppliers: PVs. Several prevailing sustainability metrics are calculated based on the CED and GWP, respectively. GWP was illustrated over an integrated time horizon of 100 years, using the impact assessment method described by IPCC 2013 GWP 100a^71,72^. We first normalize the life cycle GWP on a 1 m^2^ PV module basis, then analyze the corresponding life cycle stage breakdown. EPBT, the time needed to generate as much energy as consumed during the production stages, is an essential metric adopted widely in characterizing the energy sustainability of PV technologies. EPBT is dominated by energy embedded in raw materials and energy consumed in manufacturing products. EROI, the amount of energy expended to produce a certain amount of energy, is another critical metric proportional to the inverse of EPBT. Besides metrics that describe the energy use, the carbon emission factor or life cycle carbon emission factor, the total amount of GHG emission mainly induced from material production and PV manufacturing is also a crucial metric describing the climate change impact of the PV system. When calculating these metrics, we account for the geographical and temporal influence on input parameters, including solar irradiation, module efficiency, etc. Additional life cycle impact assessment results are presented in Supplementary Discussion 4: Life cycle impact assessment results.

### Supply chain structure and information

The trade data are collected from USITC and calculated as market shares in each offshore manufacturing case, as shown in Fig. 5 and Table 1^36^. All trade information can be obtained by using the Harmonized Tariff Schedule (HTS) code, which is the primary tool applied for data search in this study. We also refer to the ruling references on tariff classification in customs rulings online search system (CROSS) from U.S. Customs and Border Protection regarding solar PV product HTS codes. Based on the ruling references provided in CROSS, trade data are obtained based on HTS code 8541.40.6015 representing c-Si PV cells of a thickness equal to or greater than 20 mm assembled into modules or panels, and more subdivisions on the type of c-Si are not available. The trade data corresponding to c-Si PV is only available for years after 2018. Since no data are available back in 2010 and 2015 using the former HTS code representing c-Si PV commodities, we select another HTS code 8541.40.6020, that represents solar cells assembled into modules or panels without the type of solar cells specified, which are retrievable in 2010 and 2015. The corresponding trade data are the best available for offshore manufacturing cases before 2018. The percentage distributions of solar PV supply market share for the U.S. are thus calculated from the trade data. Additional information regarding supply chain structure are provided in Supplementary Note 1: Supply chain and trade data.

**Table 1:** Supply chain structure’s data sources and assumptions for offshore manufacturing cases

Case	2010 offshore manufacturing case		2015 offshore manufacturing case		2020 offshore manufacturing case	
Data source	United States International Trade Commission^81^		United States International Trade Commission^81^		United States International Trade Commission^81^	
Trade flow	General Imports		General Imports		General Imports	
Classification system	Harmonized Tariff Schedule (HTS) items		Harmonized Tariff Schedule (HTS) items		Harmonized Tariff Schedule (HTS) items	
Data to report	General First Unit of Quantity		General First Unit of Quantity		General First Unit of Quantity	
Data and years	Annual 2010		Annual 2015		Annual 2020	
HTS code	8541.40.6020 (data available for years before 2018, not available for the year 2020)		8541.40.6020 (data available for years before 2018, not available for the year 2020)		8541.40.6015 (data available for years after 2018, not available for the years 2010 and 2015)	
General Imports | Annual Data | General First Unit of Quantity	Taiwan	31,118,669	Malaysia	22,758,420	Malaysia	25,869,581
	Malaysia	21,924,800	China	22,193,576	Vietnam	14,122,856
	China	10,794,901	Taiwan	5,092,395	Thailand	11,248,003
	Germany	4,515,640	Japan	4,232,078	South Korea	6,180,956
	Japan	4,204,608	Mexico	3,592,975	China	4,059,034
	Mexico	1,414,578	South Korea	3,362,024	Singapore	1,897,098
	Switzerland	217,184	Germany	2,921,581	Cambodia	1,571,176
	Philippines	157,528	Singapore	2,270,306	Turkey	708,348
	India	117,241	Vietnam	2,080,209	Canada	633,819
	Spain	116,329	France	692,327	Taiwan	542,271
	United Kingdom	74,598	Canada	558,277	Mexico	492,055
	Hong Kong	72,254	India	504,147	India	252,049
	Norway	63,300	Philippines	371,349	Italy	239,955
	Sweden	62,151	Turkey	364,054	Philippines	134,212
	Canada	49,635	Thailand	361,106	Indonesia	58,994
	South Korea	45,467	Hungary	252,358	Burma	44,689
	Belgium	31,529	Cyprus	57,064	Japan	26,041
	Italy	18,837	Portugal	44,954	Jordan	4,280
	France	17,721	United Kingdom	33,702	Haiti	4,050
	Portugal	14,019	Hong Kong	30,414	Brazil	2,851
	Hungary	9300	Norway	25,268	Poland	2,782
	Netherlands	5560	Indonesia	11,739	Australia	2,741
	Singapore	5509	Italy	6838	Lithuania	1,097
	Austria	4443	Belize	5975	France	1,033
	Vietnam	3426	Spain	3038	Germany	588
	Argentina	2920	Netherlands	2199	Belgium	450
	South Africa	2222	Australia	1008	Georgia	260
	Czechia	1634	United Arab Em	556	United Kingdom	99
	Turkey	1323	Pakistan	250	Bangladesh	60
	Australia	822	Belgium	121	Netherlands	19
	Brazil	500	New Zealand	110	Sweden	9
	Denmark	202	Poland	54	United Arab Em	3
	New Zealand	200	Slovakia	12	Total	68,101,459
	Poland	138	Israel	8		
	Thailand	50	Estonia	5		
	Israel	38	Dominican Rep	1		
	Luxembourg	2	Denmark	1		
	Indonesia	1	Total	71,830,499		
	Total	75,069,279				
Market shares calculated from “General Imports | Annual Data | General First Unit of Quantity”	Taiwan	41%	Malaysia	32%	Malaysia	38%
	Malaysia	29%	China	31%	Vietnam	21%
	China	14%	Taiwan	7%	Thailand	17%
	Germany	6%	Japan	6%	South Korea	9%
	Japan	6%	Mexico	5%	China	6%
	Mexico	2%	South Korea	5%	Singapore	3%
	Switzerland	0%	Germany	4%	Cambodia	2%
	Philippines	0%	Singapore	3%	Turkey	1%
	India	0%	Vietnam	3%	Canada	1%
	Spain	0%	France	1%	Taiwan	1%
	United Kingdom	0%	Canada	1%	Mexico	1%
	Hong Kong	0%	India	1%	India	0%
	Norway	0%	Philippines	1%	Italy	0%
	Sweden	0%	Turkey	1%	Philippines	0%
	Canada	0%	Thailand	1%	Indonesia	0%
	South Korea	0%	Hungary	0%	Burma	0%
	Belgium	0%	Cyprus	0%	Japan	0%
	Italy	0%	Portugal	0%	Jordan	0%
	France	0%	United Kingdom	0%	Haiti	0%
	Portugal	0%	Hong Kong	0%	Brazil	0%
	Hungary	0%	Norway	0%	Poland	0%
	Netherlands	0%	Indonesia	0%	Australia	0%
	Singapore	0%	Italy	0%	Lithuania	0%
	Austria	0%	Belize	0%	France	0%
	Vietnam	0%	Spain	0%	Germany	0%
	Argentina	0%	Netherlands	0%	Belgium	0%
	South Africa	0%	Australia	0%	Georgia	0%
	Czechia	0%	United Arab Em	0%	United Kingdom	0%
	Turkey	0%	Pakistan	0%	Bangladesh	0%
	Australia	0%	Belgium	0%	Netherlands	0%
	Brazil	0%	New Zealand	0%	Sweden	0%
	Denmark	0%	Poland	0%	United Arab Em	0%
	New Zealand	0%	Slovakia	0%		
	Poland	0%	Israel	0%		
	Thailand	0%	Estonia	0%		
	Israel	0%	Dominican Rep	0%		
	Luxembourg	0%	Denmark	0%		
	Indonesia	0%				
Assumed market shares in this research analysis	Taiwan	41%	Malaysia	32%	Malaysia	38%
	Malaysia	29%	China	31%	Vietnam	21%
	China	14%	Taiwan	7%	Thailand	17%
	Germany	6%	Japan	6%	South Korea	9%
	Japan	6%	Mexico	5%	China	6%
	Mexico	2%	South Korea	5%	Singapore	3%
	Rest of the World	1%	Rest of the World	15%	Rest of the World	7%

### Energy structure

We obtain time series electricity generation by source data of each region from decades ago to recent years^39^. Typical electricity sources include coal, oil, natural gas, hydropower, nuclear, wind, etc. As the annual electricity generation increases for most regions over the years, the shares of sources also vary dramatically based on each region’s individual productivity advancements, energy demand requests, energy policy emphasis, and carbon neutrality targets. The data in 2010 and 2015 were retrieved directly, while the data used to represent the 2020 case are the closest to 2020, as shown in Supplementary Fig. 10.

The energy structures of different trading partners as well as the corresponding emissions gerneated from each region’s unit power usage have differed drastically in the past ten years, as shown in Table 2. While China’s coal demand and production capacity remained high, the government pushed to reduce emissions, improve air quality, and enhance the competitiveness of wind and solar PV in the electricity network. In Malaysia, the share of natural gas in the power mix continued to decrease by roughly 10% every five years, from 67% in 2005 to 57% in 2010, and from 47% in 2015 to 37% in 2019, responding to the policy of switching to coal and hydro fuels. Japan was one of the few countries with a decrease in power generation from 2010 to 2020 based on its energy security, economic efficiency, as well as environmental sustainability principles. Vietnam relied more on hydropower than other countries, but its coal-fired capacity was on the rise until 2019. South Korea’s energy sector was characterized by a predominance of fossil fuels, including coal, natural gas, and nuclear power, on par with Taiwan’s. Singapore, Thailand, and Mexico relied heavily on natural gas as their primary power source. In contrast, Germany relied on a wide variety of fuels, with a significant increase in the contribution of wind power from 2010 to 2020. While most regions in the world still rely on natural gas and coal as major sources of electricity, most have set goals to diversify the energy mix soon, phasing out traditional fossil fuels and increasing the share of renewables.

**Table 2:** Energy structure of solar PV importers (top six locations), power generation data sources, and emissions generated from each region’s unit power usage

Case	2010 Offshore manufacturing case			2015 Offshore manufacturing case			2020 Offshore manufacturing case		
Individual region’s power generation data source	International Energy Agency^39^			International Energy Agency^39^			International Energy Agency^39^		
	World Energy Balances 2021^82^			World Energy Balances 2021^82^			World Energy Balances 2021^82^		
Top six locations (importers to the U.S.)	Taiwan (41%)			Malaysia (32%)			Malaysia (38%)		
	Malaysia (29%)			China (31%)			Vietnam (21%)		
	China (14%)			Taiwan (7%)			Thailand (17%)		
	Germany (6%)			Japan (6%)			South Korea (9%)		
	Japan (6%)			Mexico (5%)			China (6%)		
	Mexico (2%)			South Korea (5%)			Singapore (3%)		
	Rest of the World (1%)			Rest of the World (15%)			Rest of the World (7%)		
Individual region’s electricity generation by source data (unit in GWh)	Taiwan/Chinese Taipei	Coal	122,426	Malaysia	Natural gas	69,962	Malaysia	Coal	80,633
		Natural gas	60,246		Coal	63,474		Natural gas	65,156
		Nuclear	41,629		Hydro	13,924		Hydro	26,666
		Oil	11,100		Oil	1739		Biofuels	1410
		Hydro	7255		Biofuels	751		Oil	969
		Waste	3128		Solar PV	273		Solar PV	943
		Wind	1026	China	Coal	4,108,994	Vietnam	Coal	118,806
		Biofuels	270		Hydro	1,130,270		Hydro	66,117
		Solar PV	26		Wind	1,85,766		Natural gas	42,507
	Malaysia	Natural gas	70,795		Nuclear	1,70,789		Solar PV	4818
		Coal	42,839		Natural gas	1,45,346		Biofuels	2842
		Hydro	6472		Biofuels	52,700		Oil	2213
		Oil	3670		Solar PV	39,500		Wind	722
		Biofuels	1002		Waste	11,029	Thailand	Natural gas	120,402
		Waste	8		Oil	9679		Coal	36018
	China	Coal	3,239,704		Geothermal	125		Biofuels	16,760
		Hydro	722,172		Solar thermal	29		Solar PV	4928
		Natural gas	78,063		Tide	8		Hydro	4629
		Nuclear	73,880	Taiwan/Chinese Taipei	Coal	1,17,163		Wind	3083
		Wind	44,622		Natural gas	79,009		Waste	227
		Biofuels	24,800		Nuclear	36,471		Oil	131
		Oil	14,856		Oil	11,987		Geothermal	1
		Waste	9063		Hydro	7505	South Korea	Coal	226,646
		Solar PV	699		Waste	3386		Nuclear	160,184
		Geothermal	125		Wind	1526		Natural gas	151,393
		Tide	7		Solar PV	875		Solar PV	18,248
		Solar thermal	2		Biofuels	246		Biofuels	7953
	Germany	Coal	273,457	Japan	Natural gas	4,24,299		Hydro	7148
		Nuclear	140,556		Coal	3,53,151		Oil	6337
		Natural gas	90,352		Oil	91,461		Other sources	3637
		Wind	38,547		Hydro	91,270		Wind	3153
		Biofuels	29,176		Solar PV	34803		Waste	1174
		Hydro	27,353		Other sources	20,910		Tide	457
		Solar PV	11,729		Biofuels	12,880	China	Coal	5,001,122
		Waste	11,099		Waste	12,271		Hydro	1,334,859
		Oil	8741		Nuclear	9437		Wind	471,175
		Other sources	2082		Wind	5580		Nuclear	366,247
		Geothermal	28		Geothermal	2595		Solar PV	269,718
	Japan	Natural gas	3,32,287	Mexico	Natural gas	1,86,251		Natural gas	218,242
		Coal	3,17,243		Coal	33,808		Biofuels	113,961
		Nuclear	2,88,230		Oil	31,577		Oil	10,799
		Oil	90,803		Hydro	30,815		Waste	10,301
		Hydro	90,681		Nuclear	11,577		Solar thermal	1317
		Other sources	20,853		Wind	8745		Geothermal	125
		Waste	10,953		Geothermal	6331		Tide	12
		Biofuels	9656		Biofuels	1341	Singapore	Natural gas	50,811
		Wind	4016		Solar PV	239		Waste	937
		Solar PV	3543		Waste	28		Solar PV	622
		Geothermal	2632	South Korea	Coal	2,36,586		Coal	619
	Mexico	Natural gas	1,46,994		Nuclear	1,64,762		Oil	218
		Oil	44,587		Natural gas	1,22,856		Biofuels	207
		Hydro	37,131		Oil	12518			
		Coal	32,282		Hydro	5796			
		Geothermal	6618		Solar PV	3975			
		Nuclear	5879		Biofuels	2487			
		Wind	1239		Wind	1342			
		Biofuels	728		Other sources	1216			
		Waste	48		Waste	663			
		Solar PV	31		Tide	496			
Individual region’s power mix shares calculated from “electricity generation by source data (unit in GWh)”	Taiwan/Chinese Taipei	Coal	50%	Malaysia	Natural gas	47%	Malaysia	Coal	46%
		Natural gas	24%		Coal	42%		Natural gas	37%
		Nuclear	17%		Hydro	9%		Hydro	15%
		Oil	4%		Oil	1%		Biofuels	1%
		Hydro	3%		Biofuels	1%		Oil	1%
		Waste	1%		Solar PV	0%		Solar PV	1%
		Wind	0%	China	Coal	70%	Vietnam	Coal	50%
		Biofuels	0%		Hydro	19%		Hydro	28%
		Solar PV	0%		Wind	3%		Natural gas	18%
	Malaysia	Natural gas	57%		Nuclear	3%		Solar PV	2%
		Coal	34%		Natural gas	2%		Biofuels	1%
		Hydro	5%		Biofuels	1%		Oil	1%
		Oil	3%		Solar PV	1%		Wind	0%
		Biofuels	1%		Waste	0%	Thailand	Natural gas	65%
		Waste	0%		Oil	0%		Coal	19%
	China	Coal	77%		Geothermal	0%		Biofuels	9%
		Hydro	17%		Solar thermal	0%		Solar PV	3%
		Natural gas	2%		Tide	0%		Hydro	2%
		Nuclear	2%	Taiwan/Chinese Taipei	Coal	45%		Wind	2%
		Wind	1%		Natural gas	31%		Waste	0%
		Biofuels	1%		Nuclear	14%		Oil	0%
		Oil	0%		Oil	5%		Geothermal	0%
		Waste	0%		Hydro	3%	South Korea	Coal	39%
		Solar PV	0%		Waste	1%		Nuclear	27%
		Geothermal	0%		Wind	1%		Natural gas	26%
		Tide	0%		Solar PV	0%		Solar PV	3%
		Solar thermal	0%		Biofuels	0%		Biofuels	1%
	Germany	Coal	43%	Japan	Natural gas	40%		Hydro	1%
		Nuclear	22%		Coal	33%		Oil	1%
		Natural gas	14%		Oil	9%		Other sources	1%
		Wind	6%		Hydro	9%		Wind	1%
		Biofuels	5%		Solar PV	3%		Waste	0%
		Hydro	4%		Other sources	2%		Tide	0%
		Solar PV	2%		Biofuels	1%	China	Coal	64%
		Waste	2%		Waste	1%		Hydro	17%
		Oil	1%		Nuclear	1%		Wind	6%
		Other sources	0%		Wind	1%		Nuclear	5%
		Geothermal	0%		Geothermal	0%		Solar PV	3%
	Japan	Natural gas	28%	Mexico	Natural gas	60%		Natural gas	3%
		Coal	27%		Coal	11%		Biofuels	1%
		Nuclear	25%		Oil	10%		Oil	0%
		Oil	8%		Hydro	10%		Waste	0%
		Hydro	8%		Nuclear	4%		Solar thermal	0%
		Other sources	2%		Wind	3%		Geothermal	0%
		Waste	1%		Geothermal	2%		Tide	0%
		Biofuels	1%		Biofuels	0%	Singapore	Natural gas	95%
		Wind	0%		Solar PV	0%		Waste	2%
		Solar PV	0%		Waste	0%		Solar PV	1%
		Geothermal	0%	South Korea	Coal	43%		Coal	1%
	Mexico	Natural gas	53%		Nuclear	30%		Oil	0%
		Oil	16%		Natural gas	22%		Biofuels	0%
		Hydro	13%		Oil	2%			
		Coal	12%		Hydro	1%			
		Geothermal	2%		Solar PV	1%			
		Nuclear	2%		Biofuels	0%			
		Wind	0%		Wind	0%			
		Biofuels	0%		Other sources	0%			
		Waste	0%		Waste	0%			
		Solar PV	0%		Tide	0%			
Emissions generated from 1kWh usage of medium voltage power (kg CO2 eq)	Taiwan	0.798	Malaysia	0.845	Malaysia	0.828			
	Malaysia	0.827	China	0.902	Vietnam	0.620			
	China	0.982	Taiwan	0.780	Thailand	0.647			
	Germany	0.634	Japan	0.687	South Korea	0.622			
	Japan	0.543	Mexico	0.570	China	0.832			
	Mexico	0.606	South Korea	0.655	Singapore	0.458			
Emissions generated from 1kWh usage of high voltage power (kg CO2 eq)	Taiwan	0.796	Malaysia	0.829	Malaysia	0.812			
	Malaysia	0.811	China	0.892	Vietnam	0.608			
	China	0.971	Taiwan	0.778	Thailand	0.635			
	Germany	0.638	Japan	0.690	South Korea	0.620			
	Japan	0.544	Mexico	0.555	China	0.822			
	Mexico	0.590	South Korea	0.652	Singapore	0.460			

The U.S. energy policy landscape has also varied fundamentally over time in its ability to provide a renewable, affordable, and environmentally sustainable energy system to be anticipated in the future. The U.S. fuel mix for power generation has undergone a considerable shift, with coal power declining from around 20% to 13% and renewables rapidly growing from around 20% to 38%, driven by lower costs and policy support. Policies at the state and federal levels have encouraged significant investment in renewable energy generation. The fuel mix projection assumes no consideration of long-term structural changes in electricity demand accounting for the pandemic^8^. New capacity additions come primarily from natural gas and, increasingly, renewable technologies, as generating capacities of coal and nuclear retire. Incentives for renewables and declining technology costs support intense competition with natural gas. With the share of natural gas generating remaining relatively stable and the contribution of coal and nuclear halving, renewables will more than double their share from 2020 to 2050, as shown in Table 3. Electricity demand will grow at a modest rate throughout the forecast period. The AEO projects that renewable generation will grow faster than overall electricity demand through 2050. Wind marks the main contributor to the growth in renewable generation before 2024, accounting for more than two-thirds of the growth over this period. After the production tax credit for wind energy were to be phased out at the end of 2024, solar power would account for almost three-quarters of the growth in renewable generation.

**Table 3:** Energy structure of the U.S. from 2020 with projections to 2050, power generation data sources, and emissions generated from unit power usage

Reshored manufacturing scenarios			2020	2025	2030	2035	2040	2045	2050
U.S. power generation data source			U.S. Energy Information Administration^8^						
			World Energy Projection System (2021)^83^						
U.S. Total Net Electricity Generation by Fuel (billion kWh), Reference case	Non-renewables	Natural gas	1636	1551	1562	1584	1706	1840	1953
		Nuclear	785	745	630	609	595	599	594
		Coal	774	706	696	654	620	593	593
		Liquid fuels	16	10	9	8	8	6	6
	Renewables	Solar	132	297	497	643	762	899	1,071
		Wind	343	630	673	731	748	762	790
		Hydro	283	295	295	295	294	294	294
		Geothermal	16	19	25	32	40	45	50
		Other	76	83	87	94	99	104	108
Power mix compositions calculated from “U.S. Total Net Electricity Generation by Fuel (billion kWh), Reference case”	Non-renewables	Natural gas	40%	36%	35%	34%	35%	36%	36%
		Nuclear	19%	17%	14%	13%	12%	12%	11%
		Coal	19%	16%	16%	14%	13%	12%	11%
		Liquid fuels	0%	0%	0%	0%	0%	0%	0%
	Renewables	Solar	3%	7%	11%	14%	16%	17%	20%
		Wind	8%	15%	15%	16%	15%	15%	14%
		Hydro	7%	7%	7%	6%	6%	6%	5%
		Geothermal	0%	0%	1%	1%	1%	1%	1%
		Other	2%	2%	2%	2%	2%	2%	2%
Emissions generated from 1kWh usage of medium voltage power (kg CO2 eq)			0.451	0.395	0.383	0.361	0.350	0.339	0.332
Emissions generated from 1kWh usage of high voltage power (kg CO2 eq)			0.446	0.391	0.379	0.358	0.346	0.336	0.328

### Parameters

The grid mix efficiency indicates the average primary solar energy to electricity conversion efficiency at the demand side, usually between 6% and 40% for solar cells. In this paper, the grid mix efficiency is set to be 30% based on an average thermal to electrical energy conversion efficiency^47,73^. Another grid mix efficiency of 70% is selected as an upper bound of grid efficiency for sensitivity analysis^46,47^.

The annual electricity generation is calculated as the product of performance ratio, solar irradiation, and module efficiency used for calculating carbon emission factor and EPBT. When the actual specific yield is plotted against the rated annual irradiation in the module plane, the performance ratio marks the slope of the resulting regression line^45,47^. The typical performance ratio was around 50% to 80% in 1994 and 1997 with an average of 70% and large variance, and from 2010 onwards, it is typically around 70–90% with minor variance in performance ratio compared to 1990’s^45^. We apply the default performance ratio of 80% for a utility-scale PV system^44,74^. We also selected an upper bound of 90% and a lower bound of 70% of the performance ratio for the sensitivity analysis^45,49^. Performance ratio measures the quality of a PV plant to render the proportion of energy available for export to the grid after deduction of thermal losses, conduction losses, and energy consumed in operation, making it independent of location and often described as a quality factor^75^. Therefore, we assume that the performance ratio does not differ significantly between cases and scenarios. The solar irradiation level is assumed to be 1700 kWh/m^2^/year (medium level), a high level of 2300 kWh/m^2^/year, and a low level of 1000 kWh/m^2^/year are used for the sensitivity analysis. Total solar irradiance varies slowly on decadal and longer timescales, and variation during solar cycle 21 was about 0.1%, so it is assumed that the solar irradiance does not fluctuate significantly over time between different cases and scenarios^76,77^.

The conversion efficiencies of modules made of any of the three main types of silicon wafers have generally increased over time^78,79^. The efficiencies of average commercial wafer-based silicon modules increased from about 15% in 2010 to 20% in 2020, and record efficiencies demonstrate the potential for even further efficiency enhancements at the production level, although a physical limit for silicon solar cell conversion efficiency exists^45^. Over the past decade, mainstream module efficiency increased by 0.3–0.4% absolute per year on average^32^^.^ We first assume a module efficiency of 14%, 11%, and 13% in 2010 for sc-Si, r-Si, and mc-Si technologies, respectively^33,70^. The sc-Si, r-Si, and mc-Si module efficiencies are assumed and estimated to be 17%, 13%, and 16% in 2015^6,47^. These numbers are further updated to 20.5%, 15%, and 18% in 2020 and onwards^6,47^. The efficiencies of modules sold in 2021 typically range from 17.4% (low-grade multi-crystalline cells) to 22.7% (high-performance back-contacted cells), with an estimated average of 20% for the most produced technology^32^. As the best cell efficiency so far for sc-Si is 26.7% and for mc-Si is 23.3%^80^, moving from individual wafers to full modules, there is a systematic difference between the module efficiency and the individual cell efficiency, and the cell-to-module efficiency ratio usually falls between 85% and 90%^32^. Therefore, in our sensitivity analysis, we assume the upper bound of the sc-Si module ratio to be 24%, that of r-Si to be 18%, and that of mc-Si to be 21%. Furthermore, we take into account degradation at a rate of 0.5% annually, which would result in a 2% reduction of module efficiency over 30 years^48^, and assumed the lower bound of sc-Si, r-Si, and mc-Si module efficiencies to be 18%, 13%, and 15% considering degradation in the sensitivity analysis.

### Reporting summary

Further information on research design is available in the Nature Portfolio Reporting Summary linked to this article.

## Supplementary information

Supplementary Information

Reporting Summary

## Data Availability

All data needed to evaluate the conclusions in the paper are present in the main paper, the Supplementary Information, as well as https://github.com/PEESEgroup/c-Si-PV-. The LCIA data used in this study are available in the ecoinvent database [https://ecoinvent.org/the-ecoinvent-database/] and the supply chain data used in this study are available from the United States International Trade Commission [https://dataweb.usitc.gov/].
