# Supplementary material for: Reshoring silicon photovoltaics manufacturing contributes to decarbonization and climate change mitigation
Source: Nat Commun. 2023 Mar 8;14:1274. doi: 10.1038/s41467-023-36827-z (PMC9995667; doi:10.1038/s41467-023-36827-z)
Supplement: Supplementary file 1 — Supplementary Information [file 41467_2023_36827_MOESM1_ESM.pdf]

Supplementary Materials for

Reshoring silicon photovoltaics manufacturing contributes to decarbonization and climate change mitigation

Haoyue Liang[1], Fengqi You[1,2,3*]

[1] Systems Engineering, College of Engineering,
Cornell University, Ithaca, New York 14853, USA

[2] Robert Frederick Smith School of Chemical and Biomolecular Engineering,
Cornell University, Ithaca, New York 14853, USA

[3] Cornell Atkinson Center for Sustainability,
Cornell University, Ithaca, New York 14853, USA

[*] Corresponding author. Phone: (607) 255-1162; Fax: (607) 255-9166; Email: fengqi.you@cornell.edu

This PDF file includes:

Supplementary Text

Supplementary Figures 1 to 11

Supplementary Tables 1 to 5

Supplementary Note 1: Supply chain and trade data

The trade data for solar were collected from USITC DataWeb, the premium source of U.S. merchandise trade and tariff data[1]. All trade data are compiled from official data retrieved from the U.S. Bureau of the Census, an agency within the U.S. Department of Commerce, and can be obtained monthly, quarterly, annual, or year-to-date. From USITC DataWeb, the data can be retrieved using a sophisticated querying tool, demonstrating detailed data in various classification systems, including the Harmonized Tariff Schedule (HTS), the primary tool applied for data search in this study. We first performed HTS (2021 basic revision 12) search on PV and examined article descriptions of "photosensitive semiconductor devices, including photovoltaic cells whether or not assembled into modules or made up into panels." We also referred to the ruling references on tariff classification in customs rulings online search system (CROSS) from U.S. Customs and Border Protection regarding solar module HTS codes.

Based on the ruling references provided in CROSS and our search results from USITC, we selected one HTS code that most closely represents the commodity we study. An HTS code of 8541.40.6015 representing c-Si PV cells of a thickness equal to or greater than 20 mm assembled into modules or panels clearly provided us with the trade information regarding c-Si PV that we need, although more subdivisions for the type of c-Si are not available. Since the supply chain structure was needed for cases 2010 to 2020, we set the trade flow to be general imports to the U.S. Various types of different measures of the data can be obtained, including (1) customs value defined as the price paid or payable for merchandise excluding import duties, etc., (2) units of quantity used for tariff or quota purposes, (3) cost, insurance, and freight (CIF) import value, the landed value at the first portal of arrival excluding U.S. import duties, and so on. All types of measures every year from 2010 to 2020 were selected (Supplementary Fig. 1), and the timeframe aggregation was set to monthly to gain more comprehensive insights. The exporting regions were displayed separately, while rate provision codes and districts were aggregated. The trade data corresponding to c-Si PV cells was only available for years after 2018. Since there is no data available for the 2010 case and 2015 case using the former HTS code representing c-Si PV commodities, we selected another HTS code 8541.40.6020, representing solar cells assembled into modules or panels without the type of solar cells specified. The corresponding trade data were available before 2018, which were the best available data for benchmark cases. The maps with HTS code and the PV panel import flows are presented in Supplementary Fig. 2.

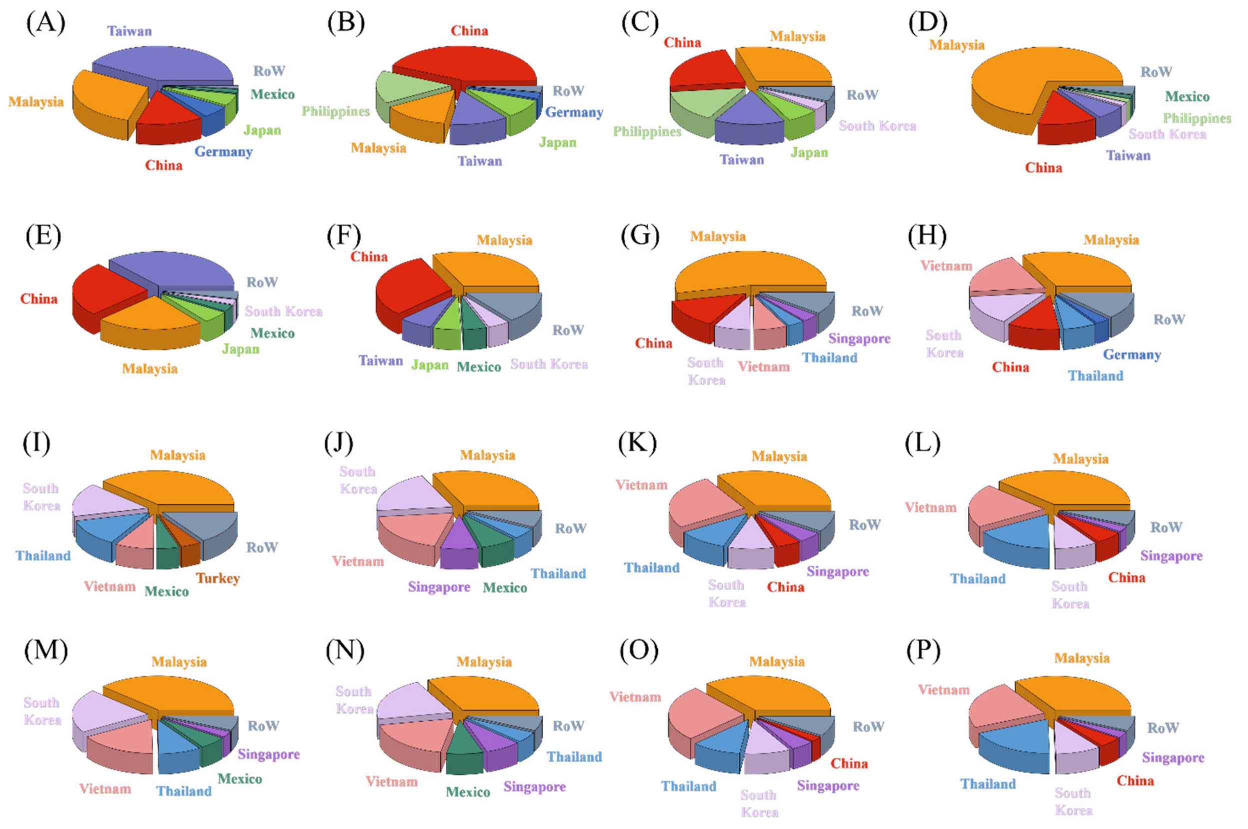

Supplementary Fig. 1 Comparison of supply chain of c-Si PVs shipped from global regions to the U.S. from 2010 to 2020 with different units and HTS code, (A)-(I) indicates supply chain structure derived from general first unit of quantity data in 2010-2018 using HTS code 8541.40.6020, (J)-(L) indicates supply chain structure derived from general first unit of quantity data in 2018-2020 using HTS code 8541.40.6015, (M) indicates supply chain structure derived from general second of quantity data in 2018 using HTS code 8541.40.6020, (N)-(P) indicates supply chain structure derived from general second of quantity data in 2018 using HTS code 8541.40.6015.

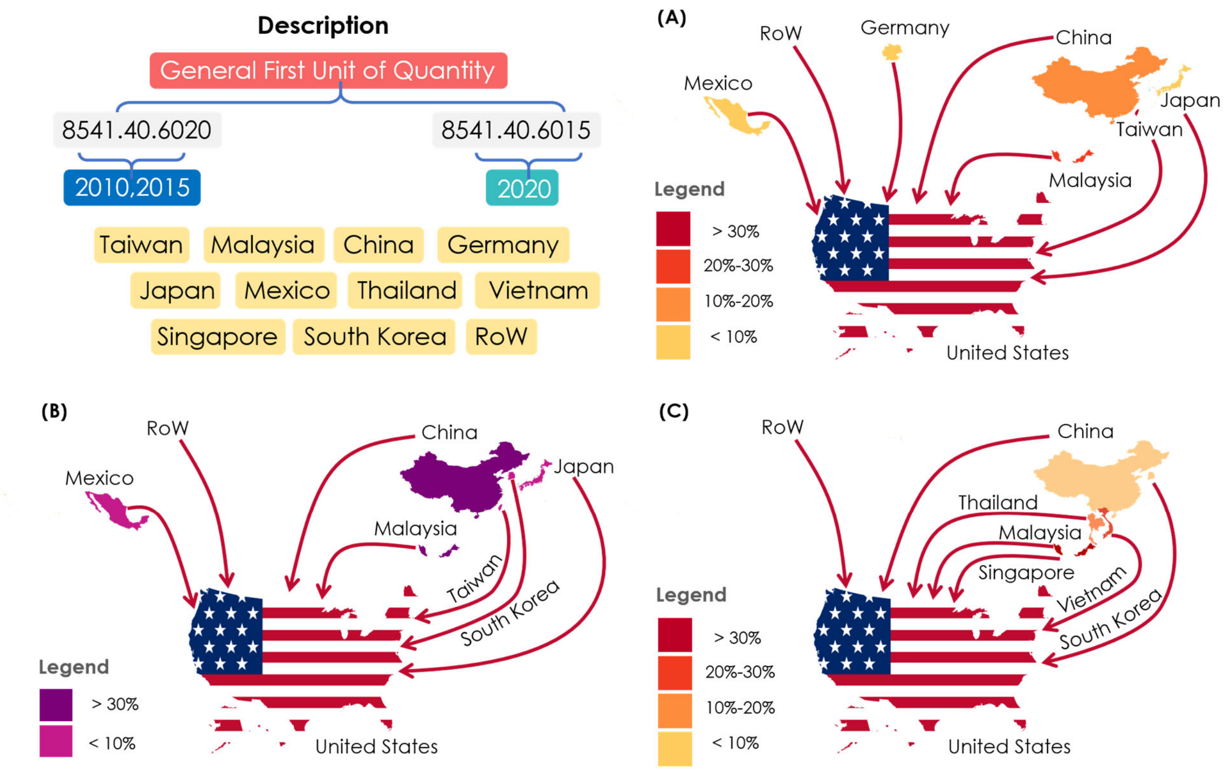

Supplementary Fig. 2 Supply chain of c-Si PVs shipped from global regions to the U.S. in 2010 and 2015 from HTS code 8541.40.6020 (as shown in panel (A) corresponding to 2010 and panel (B) representing 2015), and in 2020 from HTS code 8541.40.6015 (as shown in panel (C)), with market shares color-coded.

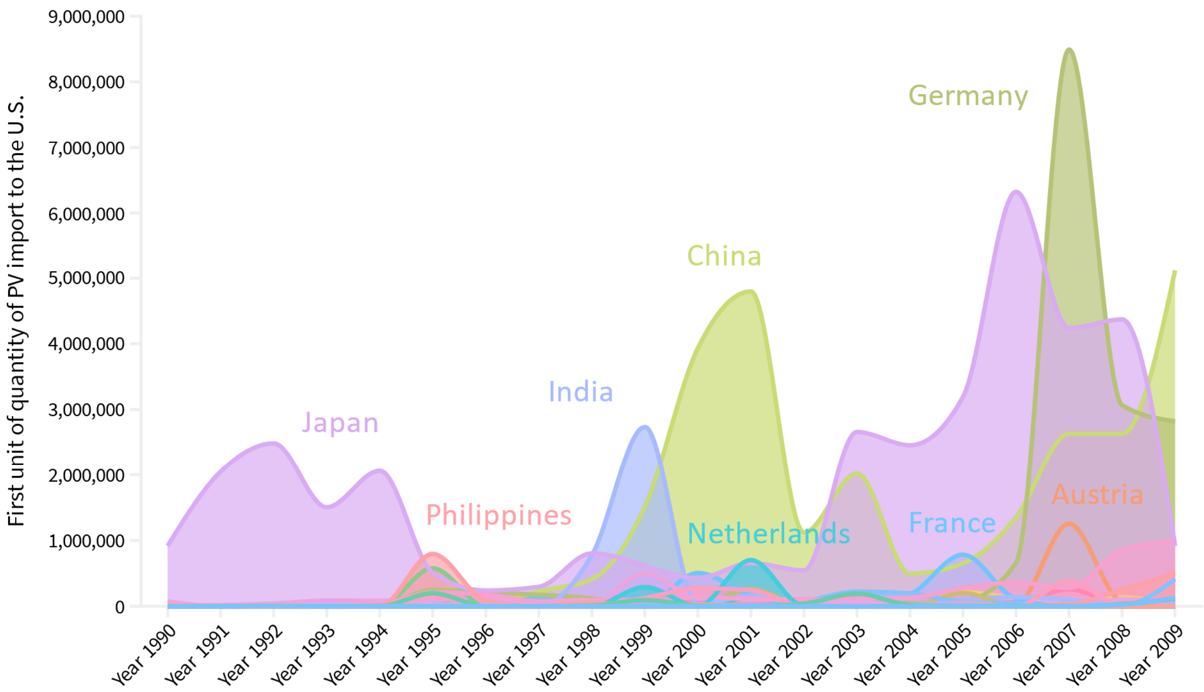

Supplementary Fig. 3 Supply chain of PVs shipped from global trading partners to the U.S. from 1990 to 2009. The supply chain structure is derived from the general first unit of quantity data from 1990 to 2009 using HTS code 8541.40.6020.

Supplementary Discussion 1: Manufacturing vs operations and maintenance vs end-of-life treatment

The GHG emissions from the use stage contributes to less than 0.20% of PV lifetime emissions, and those from the end-of-life management stage contributes to less than 0.41%, as shown in Supplementary Fig. 4. The energy use from the use stage contributes to less than 2.0% of lifetime energy use, and that from the end-of-life management stage contributes to less than 3.0%. Since most carbon emissions occur in the upstream manufacturing process, and contributions of emissions from the use stage and the disposal stage are generally low.

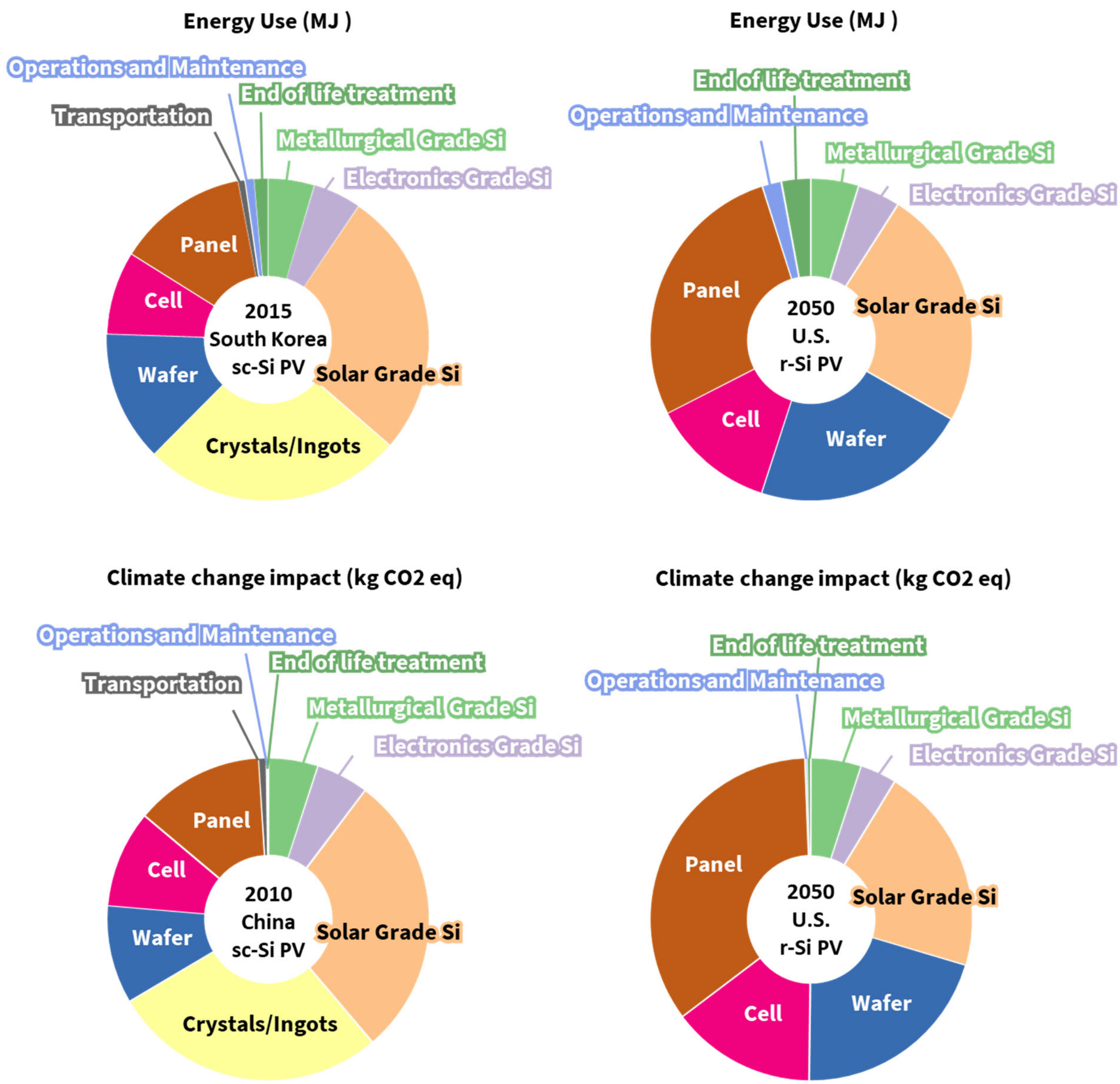

Supplementary Fig. 4 Energy use and climate change impact from the manufacturing stage compared against the operations and maintenance stage, and the end-of-life treatment stage. Among all manufacturing locations, PV technologies, and temporal variations, South Korea in 2015 used 5244.6 MJ

energy to manufacture sc-Si PV, the highest among all. On the other hand, among all cases and scenarios, the U.S. is projected to use the least amount of energy to manufacture r-Si PV panels in 2050. Despite the dramatic difference between the highest and lowest energy use from the PV manufacturing stage discussed in this study, the energy use of the operations and maintenance stage contributes to less than 2.0% of lifetime energy use, and the end-of-life treatment stage contributes to less than 3.0%. Similarly, the GHG emissions from manufacturing sc-Si PV in China in 2010 are the highest, while the emissions from manufacturing r-Si PV panels in the U.S. in 2050 are projected to be the lowest. Regardless of the cases and scenarios, the climate change impact from the use stage contributes to less than 0.20% of overall emissions, and the disposal stage contributes to less than 0.41%.

Supplementary Discussion 2: Counterfactual scenario in 2035

The energy use and climate change impact in 2035 compared to in 2020 if the trading partners and their supply shares remain unchanged have also been investigated. The comparison between the 2020 offshore manufacturing case and the 2035 counterfactual scenario provides insights into how the dynamic energy mix influences the energy and environmental impacts of solar panel manufactured at outsourced locations, given that the supplies from trading partners remain unchanged. The counterfactual scenario in 2035 show that if the U.S. continues to rely on the same trading partners as in 2020, the energy use and GHG emissions from panel manufacutirng would be reduced by 4% and 13% as shown in Supplementary Fig. 5.

From Supplementary Fig. 1 for data regarding solar panel trading partners and their market shares from 2010 to 2020 and Supplementary Fig. 3 showcasing the fluctuating data regarding panel importers from 1990 to 2009, the supply chain structure for the past 30 years is highly dynamic with continuous fluctuations. The supply chain structure can be easily influenced by geopolitical issues. Due to the uncertainties arising from these issues, long term projections of the economic and supply chain structures could have large error bars. As a result, following the trajectory and current policy agenda, multiple likely reshored manufacturing scenarios are the main focus in this study.

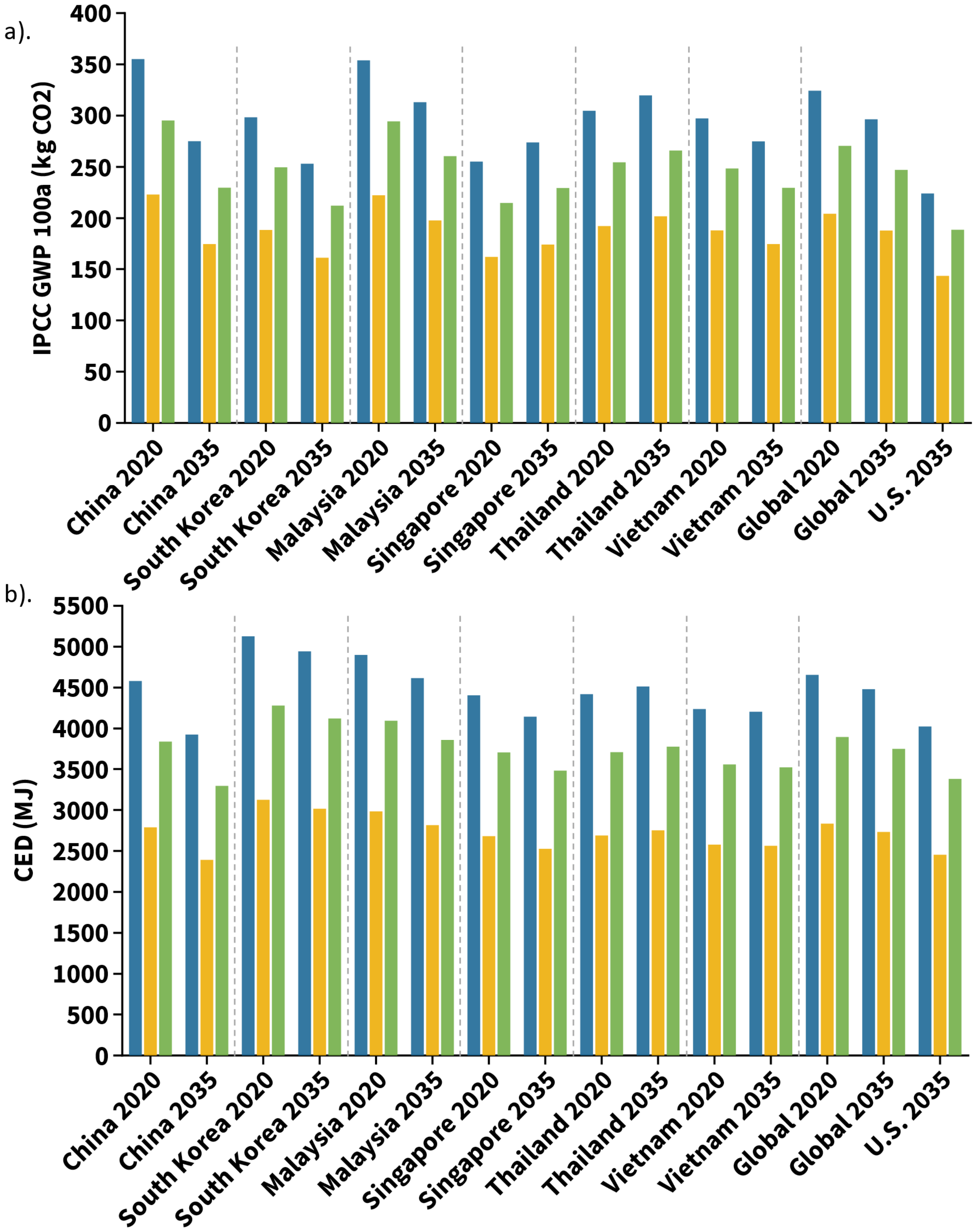

Supplementary Fig. 5 Energy use and climate change impact results of the 2035 counterfactual scenario using the current supply chain structure (trading partners in the 2020 case: China, South Korea, Malaysia, Singapore, Thailand, and Vietnam) and the global energy mix projection in 2035, in comparison with the

2020 offshore manufacturing case and the 2035 reshored manufacturing scenario. The figures show reductions in climate change impact (panel **a**) and energy use (panel **b**) in 2035 compared to in 2020 if the trading partners and their supply shares remain unchanged. Global offshore manufacturing scenario in 2035 (counterfactual scenario) results in a less than 4% reduction in energy use and a less than 9% reduction in GHG emissions, compared to the 2020 offshore case. However, reshored manufacturing in 2035 results in a 13% reduction in energy use, as well as a 30% reduction in emissions, compared to the same 2020 offshore case.

Supplementary Discussion 3: Partially reshored scenarios in 2035

Apart from the fully reshored scenario and counterfactual scenario in 2035, partially reshored scenarios are also taken into account in this study. There is a chance that the U.S. might not be fully equipped to bring PV manufacturing back completely[2,3]. Instead of fully reshoring PV panel production, achieving a greater share of U.S. production that is less than 100% is also possible. We thus study the energy and environmental impacts of three examples of partially reshored scenarios, of which the U.S. supplies 25%, 50%, and 75% of its own PV panels, while relying on the remaining 75%, 50%, and 25% supplies from external sources.

Based on the quantitative results as shown in Supplementary Fig. 6 and Supplementary Fig. 7, party reshored scenario a (25% production from the U.S. and 75% production from global regions) results in 2.5% lower energy consumption and 6% lower emissions, while party reshored scenario b (50% production from the U.S. and 50% production from global regions) results in 5% lower energy use and 12% lower climate change impact, and partially reshored scenario c (75% production from the U.S. and 25% production from global regions) results in less than 8% reduced energy demand and 18% reduced carbon emissions, all compared to the counterfactual scenario (100% production from global regions and no domestic manufacturing) in 2035. Partially reshored scenarios align with the climate change mitigation goal and sustainability target more than the counterfactual scenario in 2035. However, the fully reshored scenario still demonstrates the best energy and environmental performances. Compared with the fully reshored scenario, partially reshored scenarios result in 2.7% to 8.5% higher energy use as well as 7.7% to 24.2% higher carbon emissions.

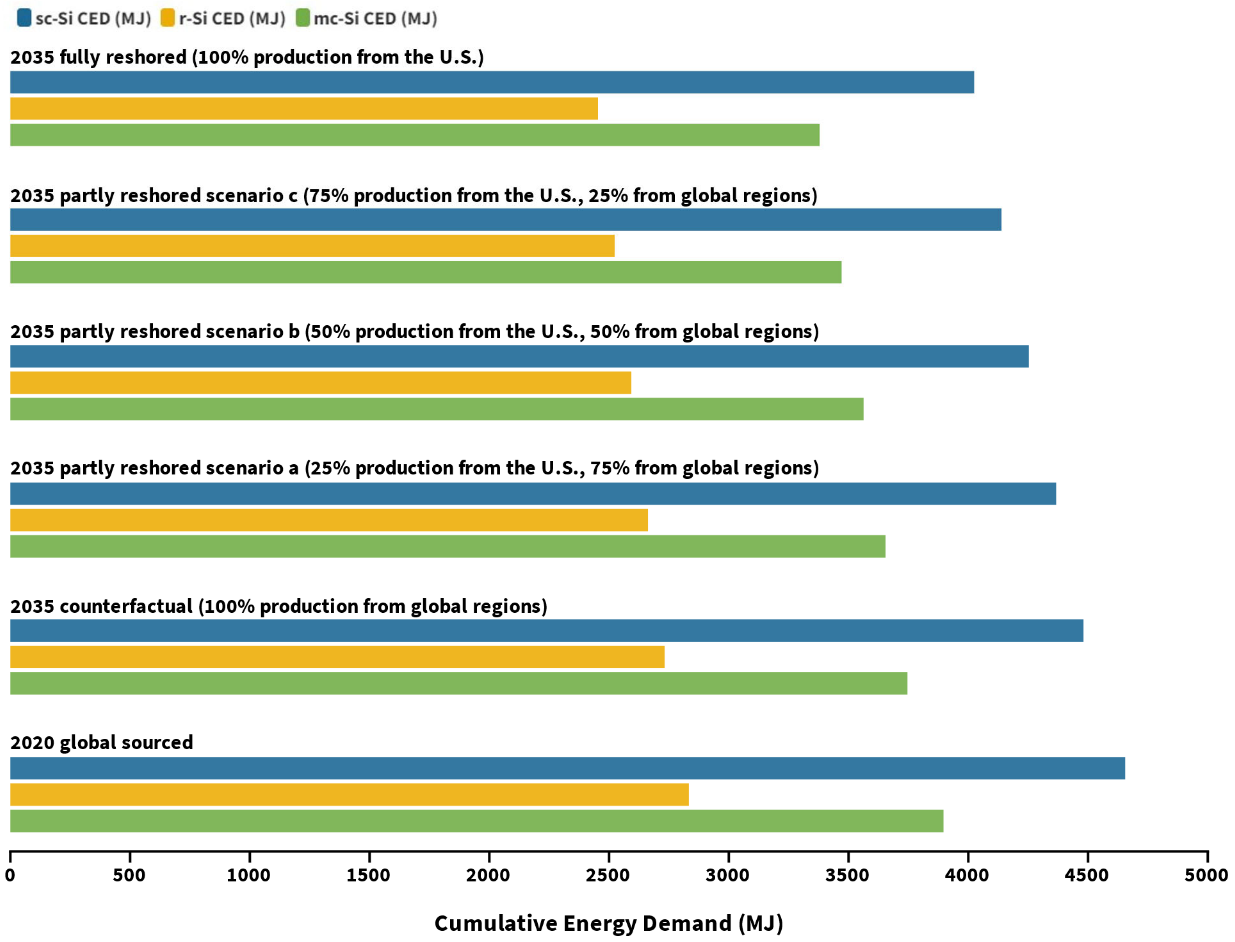

Supplementary Fig. 6 Energy use of partially reshored scenarios in comparison with fully reshored and global sourced scenarios in 2035. It is also likely that instead of achieving fully reshored PV panel manufacturing in the U.S., there might be a greater share of U.S. production that is less than 100%. Here, we formulate three example scenarios of which the U.S. partially reshores PV panel manufacturing (2035 partially reshored scenario a relies on 25% domestic production, 2035 partially reshored scenario b relies on 50% domestic production, and 2035 partially reshored scenario c relies on 75% domestic production. The rest of the PV panels come from global supplies. Partially reshored scenarios result in overall lower energy use than global sourced scenario but higher energy use than fully reshored scenario in 2035.

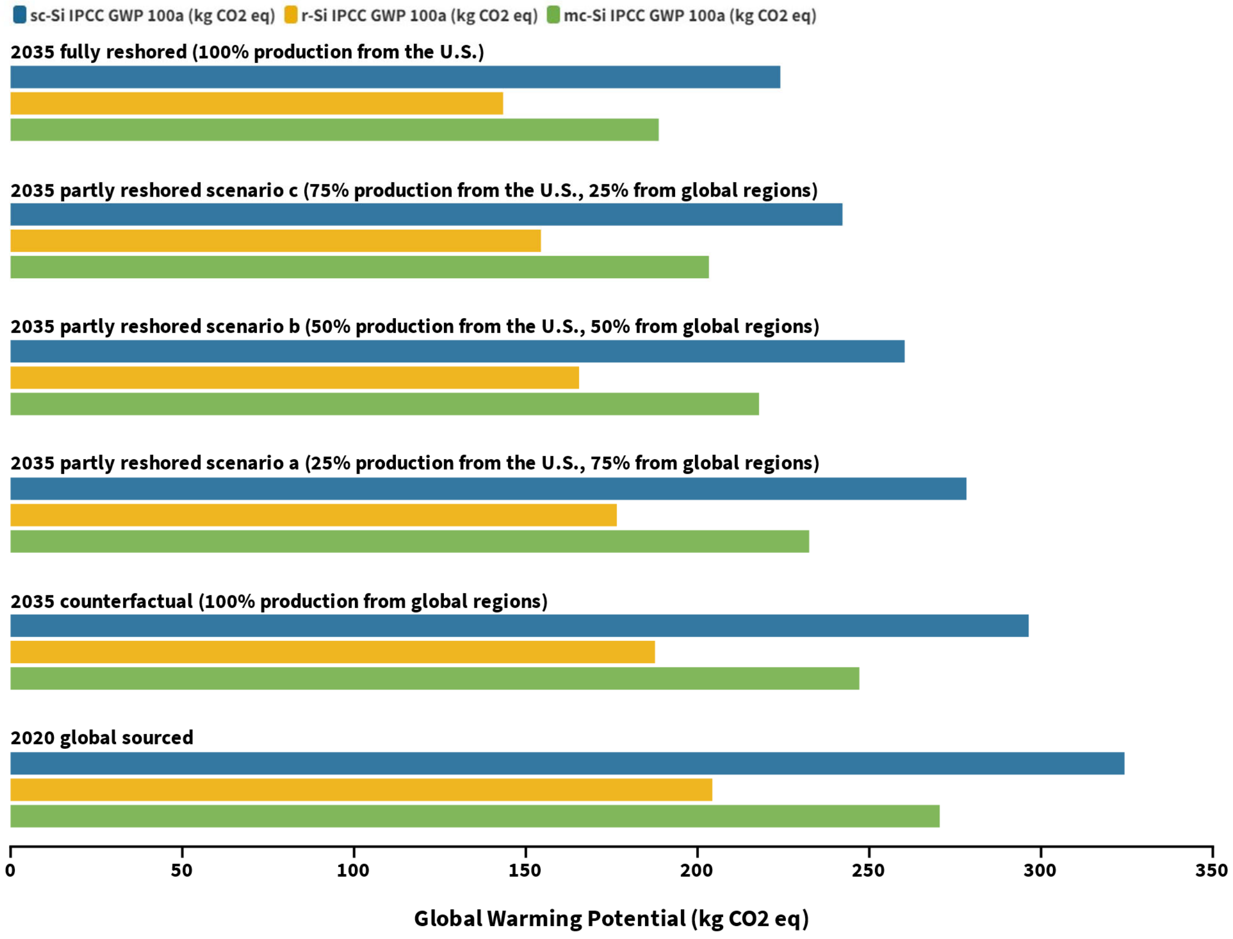

Supplementary Fig. 7 Climate change impacts of partially reshored scenarios in comparison with fully reshored and global sourced scenarios in 2035. It is also likely that instead of achieving fully reshored PV panel manufacturing in the U.S., there might be a greater share of U.S. production that is less than 100%. Here, we formulate three example scenarios of which the U.S. partially reshores PV panel manufacturing (2035 partially reshored scenario a relies on 25% domestic production, 2035 partially reshored scenario b relies on 50% domestic production, and 2035 partially reshored scenario c relies on 75% domestic production. The rest of the PV panels come from global supplies. Partially reshored scenarios result in overall lower carbon emissions than global sourced scenario but higher carbon emissions than fully reshored scenario in 2035.

Supplementary Methods 1: Life cycle inventory and life cycle stages

In this work, we aim to evaluate the “cradle-to-site” climate change and energy impacts of reshored c-Si PV panel manufacturing to assess if the act of bringing manufacturing back home aligns with the climate target. The system boundary of the life cycle of c-Si PVs consists of several stages, from raw material acquisition to solar module production, as shown in Supplementary Fig. 8. We include all major and minor manufacturing and construction materials, from wet wood chips in quartz mining to low-iron solar

glass in module production, in the inventory (Supplementary Fig. 9). To enable a fair comparison of modules in different cases and scenarios regarding material inputs, energy consumption, and emissions, we define the system boundary to be from silica sand mining to panel manufacturing to shipping panels to the U.S. for all for consistency. In this study, the overarching functional unit, typically defined in terms of a unit quantity of product, is set to be 1 m^2 of the solar module according to the previous literature[4,5], which is helpful to capture the changes in energy and environmental profiles proportional to PV size directly over time. We note that the end-of-life phase is excluded from the system boundary following assumptions in existing literature accounting for a lack of data[6], as there has been insufficient data on the disposal phase as well as the balance of plants[7].

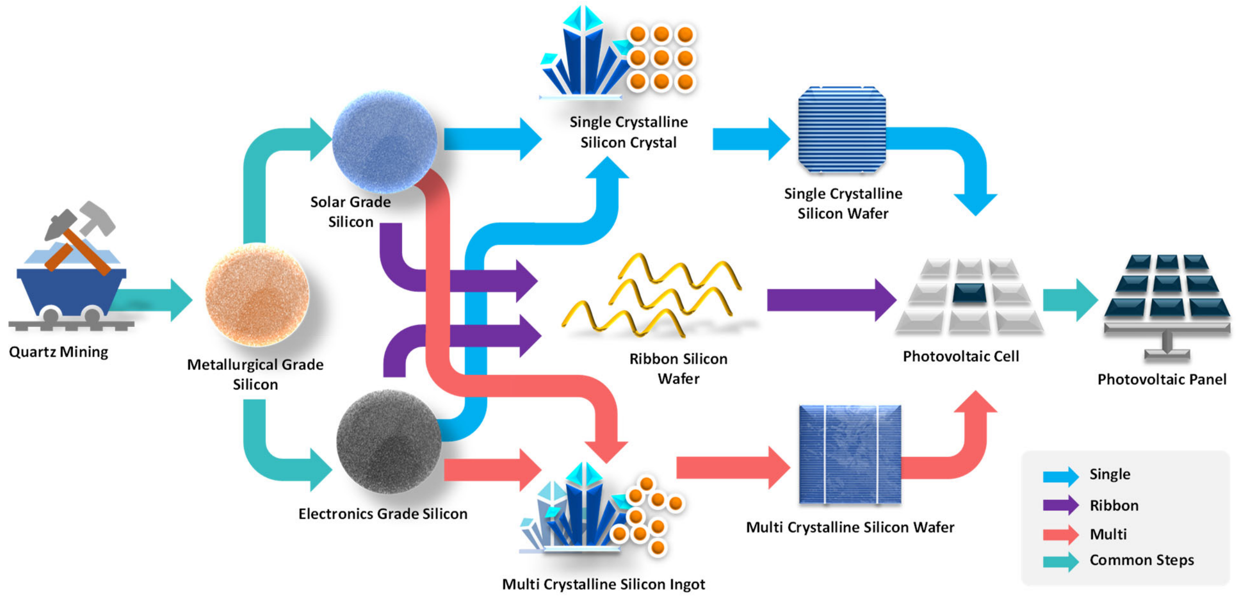

Supplementary Fig. 8 The flow diagram of manufacturing PV modules based on three types of technologies (sc-Si, r-Si, mc-Si). Flows of each type of technology are color-coded. The production of three types of silicon materials all requires the use of solar grade silicon and electronics grade silicon.

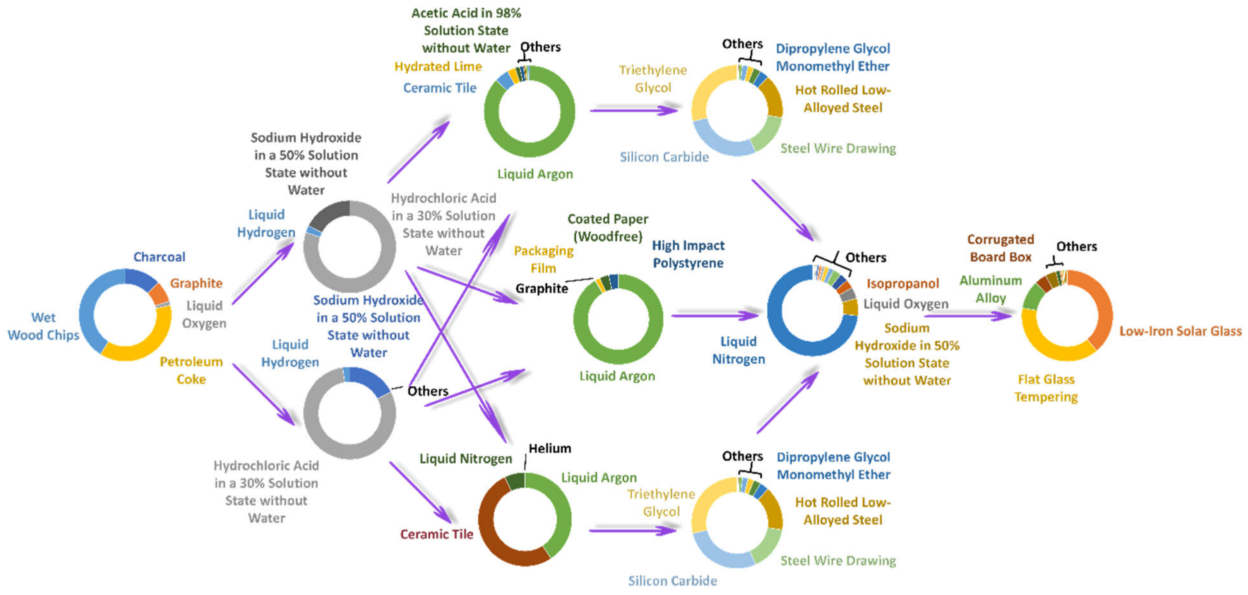

Supplementary Fig. 9 Major LCI beside from silicon materials involved in manufacturing stages from raw material mining to c-Si module manufacturing.

Table S1 presents the energy and material inventory needed under various manufacturing stages to produce one squared meter of the c-Si PV panel. In order to generate one functional unit of PV panel, the amount of energy and material inventory input and output at the panel manufacturing stage are the same regardless of the c-Si PV technology. At the cell manufacturing stage, the inventory input and output are also similar regardless of c-Si PV type, except that the amount of wafer needed to generate the same size of a cell is different for r-Si PV compared to sc-Si PV and mc-Si PV. At the wafer manufacturing stage, r-Si PV technology utilizes solar and electronics grade silicon directly to manufacture wafers. On the other hand, sc-Si PV and mc-Si PV technologies need to undergo crystal formation or ingot formation stage before proceeding to the wafer manufacturing stage.

Table S1. Material and energy inventory of 1 m^2 of the three types of c-Si PV panel.

sc-Si				r-Si				mc-Si			
Process	Flow	Amount	Unit	Process	Flow	Amount	Unit	Process	Flow	Amount	Unit
Panel manufacturing				Panel manufacturing				Panel manufacturing			
input	1-propanol	0.0081386	kg	input	1-propanol	0.0081386	kg	input	1-propanol	0.0081386	kg
	acetone, liquid	0.012959	kg		acetone, liquid	0.012959	kg		acetone, liquid	0.012959	kg
	aluminum alloy, AlMg3	2.6294	kg		aluminum alloy, AlMg3	2.6294	kg		aluminum alloy, AlMg3	2.6294	kg
	brazing solder, cadmium free	0.0087647	kg		brazing solder, cadmium free	0.0087647	kg		brazing solder, cadmium free	0.0087647	kg
	copper, cathode	0.11269	kg		copper, cathode	0.11269	kg		copper, cathode	0.11269	kg
	corrugated board box	1.0956	kg		corrugated board box	1.0956	kg		corrugated board box	1.0956	kg
	electricity, medium voltage	4.7107	kWh		electricity, medium voltage	4.7107	kWh		electricity, medium voltage	4.7107	kWh
	ethylvinylacetate, foil	1.0017	kg		ethylvinylacetate, foil	1.0017	kg		ethylvinylacetate, foil	1.0017	kg
	glass fiber reinforced plastic, polyamide, injection molded	0.18781	kg		glass fiber reinforced plastic, polyamide, injection molded	0.18781	kg		glass fiber reinforced plastic, polyamide, injection molded	0.18781	kg
	heat, district or industrial, natural gas	4.86639	MJ		heat, district or industrial, natural gas	4.86639	MJ		heat, district or industrial, natural gas	4.86639	MJ
	lubricating oil	0.0016069	kg		lubricating oil	0.0016069	kg		lubricating oil	0.0016069	kg

	methanol	0.0021556	Kg		methanol	0.0021556	kg		methanol	0.0021556	kg
	nickel, class 1	1.63E-04	Kg		nickel, class 1	1.63E-04	kg		nickel, class 1	1.63E-04	kg
	photovoltaic cell, single-Si wafer	0.93241	m2		photovoltaic cell, ribbon-Si	0.93241	m2		photovoltaic cell, multi-Si wafer	0.93241	m2
	photovoltaic panel factory	4.00E-06	Item(s)		photovoltaic panel factory	4.00E-06	Item(s)		photovoltaic panel factory	4.00E-06	Item(s)
	polyethylene terephthalate, granulate, amorphous	0.37297	kg		polyethylene terephthalate, granulate, amorphous	0.37297	kg		polyethylene terephthalate, granulate, amorphous	0.37297	kg
	polyvinyl fluoride, film	0.1104	kg		polyvinyl fluoride, film	0.1104	kg		polyvinyl fluoride, film	0.1104	kg
	silicone product	0.12195	kg		silicone product	0.12195	kg		silicone product	0.12195	kg
	solar glass, low iron	10.079	kg		solar glass, low iron	10.079	kg		solar glass, low iron	10.079	kg
	tap water	21.286	kg		tap water	21.286	kg		tap water	21.286	kg
	tempering, flat glass	10.079	kg		tempering, flat glass	10.079	kg		tempering, flat glass	10.079	kg
	vinyl acetate	0.0016434	kg		vinyl acetate	0.0016434	kg		vinyl acetate	0.0016434	kg
	wire drawing, copper	0.11269	kg		wire drawing, copper	0.11269	kg		wire drawing, copper	0.11269	kg
output	municipal solid waste	0.03	kg	output	municipal solid waste	0.03	kg	output	municipal solid waste	0.03	kg
	photovoltaic panel, single-Si wafer	1	m2		photovoltaic panel, ribbon-Si	1	m2		photovoltaic panel, multi-Si wafer	1	m2
	waste mineral oil	1.61E-03	kg		waste mineral oil	1.61E-03	kg		waste mineral oil	1.61E-03	kg
	waste plastic, mixture	1.6861	kg		waste plastic, mixture	1.6861	kg		waste plastic, mixture	1.6861	kg
	waste polyvinyl fluoride	0.1104	kg		waste polyvinyl fluoride	0.1104	kg		waste polyvinyl fluoride	0.1104	kg
	wastewater, from residence	0.021286	m3		wastewater, from residence	0.021286	m3		wastewater, from residence	0.021286	m3
	Water	0.0031929	m3		Water	0.0031929	m3		Water	0.0031929	m3
Cell manufacturing				Cell manufacturing				Cell manufacturing			
input	acetic acid, without water, in 98% solution state	0.0026	kg	input	acetic acid, without water, in 98% solution state	0.0026	kg	input	acetic acid, without water, in 98% solution state	0.0026	kg
	ammonia, anhydrous, liquid	0.0063	kg		ammonia, anhydrous, liquid	0.0063	kg		ammonia, anhydrous, liquid	0.0063	kg
	argon, liquid	0.0239	kg		argon, liquid	0.0239	kg		argon, liquid	0.0239	kg
	calcium chloride	0.0201	kg		calcium chloride	0.0201	kg		calcium chloride	0.0201	kg
	electricity, medium voltage	28.1989	kWh		electricity, medium voltage	28.1989	kWh		electricity, medium voltage	28.1989	kWh
	ethanol, without water, in 99.7% solution state, from ethylene	0.0006	kg		ethanol, without water, in 99.7% solution state, from ethylene	0.0006	kg		ethanol, without water, in 99.7% solution state, from ethylene	0.0006	kg
	heat, district or industrial, natural gas	4	MJ		heat, district or industrial, natural gas	4	MJ		heat, district or industrial, natural gas	4	MJ
	heat, district or industrial, other than natural gas	1.0311	MJ		heat, district or industrial, other than natural gas	1.0311	MJ		heat, district or industrial, other than natural gas	1.0311	MJ
	hydrochloric acid, without water, in 30% solution state	0.0425	kg		hydrochloric acid, without water, in 30% solution state	0.0425	kg		hydrochloric acid, without water, in 30% solution state	0.0425	kg
	hydrogen fluoride	0.0352	kg		hydrogen fluoride	0.0352	kg		hydrogen fluoride	0.0352	kg
	isopropanol	0.0736	kg		isopropanol	0.0736	kg		isopropanol	0.0736	kg
	metallization paste, back side	0.0046	kg		metallization paste, back side	0.0046	kg		metallization paste, back side	0.0046	kg
	metallization paste, back side, aluminum	0.067	kg		metallization paste, back side, aluminum	0.067	kg		metallization paste, back side, aluminum	0.067	kg
	metallization paste, front side	0.0069	kg		metallization paste, front side	0.0069	kg		metallization paste, front side	0.0069	kg
	nitric acid, without water, in 50% solution state	0.0249	kg		multi-Si wafer, ribbon	1.0026	m2		multi-Si wafer	0.9884	m2
	nitrogen, liquid	1.7279	kg		nitric acid, without water, in 50% solution state	0.0249	kg		nitric acid, without water, in 50% solution state	0.0249	kg
	oxygen, liquid	0.095	kg		nitrogen, liquid	1.7279	kg		nitrogen, liquid	1.7279	kg
	phosphoric acid, fertilizer grade, without water, in 70% solution state	0.0072	kg		oxygen, liquid	0.095	kg		oxygen, liquid	0.095	kg
	phosphoryl chloride	0.0015	kg		phosphoric acid, fertilizer grade, without	0.0072	kg		phosphoric acid, fertilizer grade, without	0.0072	kg

					water, in 70% solution state				water, in 70% solution state		
	photovoltaic cell factory	0	Item (s)		phosphoryl chloride	0.0015	kg		phosphoryl chloride	0.0015	kg
	polystyrene, expandable	0.0004	kg		photovoltaic cell factory	0	Item (s)		photovoltaic cell factory	0	Item (s)
	silicone product	0.0011	kg		polystyrene, expandable	0.0004	kg		polystyrene, expandable	0.0004	kg
	single-Si wafer, photovoltaic	0.9884	m2		silicone product	0.0011	kg		silicone product	0.0011	kg
	sodium hydroxide, without water, in 50% solution state	0.1464	kg		sodium hydroxide, without water, in 50% solution state	0.1464	kg		sodium hydroxide, without water, in 50% solution state	0.1464	kg
	sodium silicate, spray powder, 80%	0.0697	kg		sodium silicate, spray powder, 80%	0.0697	kg		sodium silicate, spray powder, 80%	0.0697	kg
	solvent, organic	0.0013	kg		solvent, organic	0.0013	kg		solvent, organic	0.0013	kg
	tetrafluoroethylene	0.0029	kg		tetrafluoroethylene	0.0029	kg		tetrafluoroethylene	0.0029	kg
	titanium dioxide	0	kg		titanium dioxide	0	kg		titanium dioxide	0	kg
	water, completely softened	127.9733	kg		water, completely softened	127.9733	kg		water, completely softened	127.9733	kg
	Water, cooling, unspecified natural origin	0.931	m3		Water, cooling, unspecified natural origin	0.931	m3		Water, cooling, unspecified natural origin	0.931	m3
output	Aluminum	0.0007	kg	output	Aluminum	0.0007	kg	output	Aluminum	0.0007	kg
	Ethane, hexafluoro-, HFC-116	0.0001	kg		Ethane, hexafluoro-, HFC-116	0.0001	kg		Ethane, hexafluoro-, HFC-116	0.0001	kg
	Hydrogen chloride	0.0002	kg		Hydrogen chloride	0.0002	kg		Hydrogen chloride	0.0002	kg
	Hydrogen fluoride	0	kg		Hydrogen fluoride	0	kg		Hydrogen fluoride	0	kg
	Lead	0.0007	kg		Lead	0.0007	kg		Lead	0.0007	kg
	Methane, tetrafluoro-, R-14	0.0002	kg		Methane, tetrafluoro-, R-14	0.0002	kg		Methane, tetrafluoro-, R-14	0.0002	kg
	Nitrogen oxides	0	kg		Nitrogen oxides	0	kg		Nitrogen oxides	0	kg
	NMVOC, non-methane volatile organic compounds, unspecified origin	0.1805	kg		NMVOC, non-methane volatile organic compounds, unspecified origin	0.1805	kg		NMVOC, non-methane volatile organic compounds, unspecified origin	0.1805	kg
	Particulates, < 2.5 um	0.0025	kg		Particulates, < 2.5 um	0.0025	kg		Particulates, < 2.5 um	0.0025	kg
	photovoltaic cell, single-Si wafer	0.9324	m2		photovoltaic cell, ribbon-Si	0.9324	m2		photovoltaic cell, multi-Si wafer	0.9324	m2
	Silicon	0.0001	kg		Silicon	0.0001	kg		Silicon	0.0001	kg
	Silver	0.0007	kg		Silver	0.0007	kg		Silver	0.0007	kg
	Sodium	0	kg		Sodium	0	kg		Sodium	0	kg
	Tin	0.0007	kg		Tin	0.0007	kg		Tin	0.0007	kg
	waste, from silicon wafer production, inorganic	0.2571	kg		waste, from silicon wafer production, inorganic	0.2571	kg		waste, from silicon wafer production, inorganic	0.2571	kg
	wastewater from PV cell production	0.2026	m3		wastewater from PV cell production	0.2026	m3		wastewater from PV cell production	0.2026	m3
	Water	0.8564	m3		Water	0.8564	m3		Water	0.8564	m3
wafer manufacturing				wafer manufacturing				wafer manufacturing			
input	wafer factory	0	Item (s)	input	argon, liquid	5.2237	kg	input	wafer factory	0	Item (s)
	acrylic binder, without water, in 34% solution state	0.002	kg		electricity, medium voltage	42.4108	kWh		acetic acid, without water, in 98% solution state	0.0385	kg
	hydrochloric acid, without water, in 30% solution state	0.0027	kg		graphite	0.0066	kg		acrylic binder, without water, in 34% solution state	0.002	kg
	tap water	0.0059	kg		packaging film, low density polyethylene	0.1003	kg		alkylbenzene sulfonate, linear, petrochemical	0.2372	kg
	brass	0.0074	kg		paper, woodfree, coated	0.1905	kg		brass	0.0074	kg
	glass wool mat	0.0099	kg		polystyrene, high impact	0.2005	kg		dipropylene glycol monomethyl ether	0.2965	kg
	sodium hydroxide, without water, in 50% solution state	0.0148	kg		silicon, electronics grade	0.108	kg		glass wool mat	0.0099	kg
	acetic acid, without water, in 98% solution state	0.0385	kg		silicon, solar grade	0.634	kg		hydrochloric acid, without water, in 30% solution state	0.0027	kg
	packaging film, low density polyethylene	0.0988	kg	output	AOX, Adsorbable Organic Halogen as Cl	0.0005	kg		packaging film, low density polyethylene	0.0988	kg

	paper, woodfree, coated	0.1878	kg
	polystyrene, high impact	0.1977	kg
	alkylbenzene sulfonate, linear, petrochemical	0.2372	kg
	dipropylene glycol monomethyl ether	0.2965	kg
	silicon, single crystal, Czochralski process, photovoltaics	1.0623	kg
	steel, low-alloyed, hot rolled	1.4653	kg
	wire drawing, steel	1.4726	kg
	silicon carbide	2.5994	kg
	triethylene glycol	2.6784	kg
	water, completely softened	64.243	kg
	electricity, medium voltage	7.9068	kWh
	heat, district or industrial, natural gas	3.5581	MJ
output	AOX, Adsorbable Organic Halogen as Cl	0.0005	kg
	BOD5, Biological Oxygen Demand	0.0292	kg
	COD, Chemical Oxygen Demand	0.0292	kg
	Copper, ion	0.0001	kg
	DOC, Dissolved Organic Carbon	0.011	kg
	Nickel, ion	0.0001	kg
	Nitrogen	0.0098	kg
	Phosphate	0.0005	kg
	single-Si wafer, photovoltaic	0.9884	m2
	TOC, Total Organic Carbon	0.011	kg
	waste, from silicon wafer production	0.1087	kg
	Water	0.0642	m3
sc-Si crystal productio n			
input	acetic acid, without water, in 98% solution state	0.1147	kg
	acetone, liquid	0.0521	kg
	argon, liquid	6.1553	kg
	ceramic tile	0.3574	kg
	electricity, medium voltage	90.931 5	kWh
	heat, district or industrial, natural gas	65.225 9	MJ
	hydrogen fluoride	0.0538	kg
	lime, hydrated, packed	0.2029	kg
	nitric acid, without water, in 50% solution state	0.1006	kg
	silicon, electronics grade	0.1654	kg
	silicon, solar grade	0.9712	kg
	silicone factory	0	Item (s)
	sodium hydroxide, without water, in 50% solution state	0.0441	kg
	tap water	99.932 2	kg
	Water, cooling, unspecified natural origin	2.4783	m3

BOD5, Biological Oxygen Demand	0.0296	kg
COD, Chemical Oxygen Demand	0.0296	kg
Copper, ion	0.0001	kg
DOC, Dissolved Organic Carbon	0.0111	kg
multi-Si wafer, ribbon	1.0026	m2
Nickel, ion	0.0001	kg
Nitrogen	0.01	kg
Phosphate	0.0005	kg
TOC, Total Organic Carbon	0.0111	kg
waste, from silicon wafer production	0.007	kg

	paper, woodfree, coated	0.1878	kg
	polystyrene, high impact	0.1977	kg
	silicon carbide	2.5994	kg
	silicon, multi-Si, casted	1.1269	kg
	sodium hydroxide, without water, in 50% solution state	0.0148	kg
	steel, low-alloyed, hot rolled	1.4653	kg
	tap water	0.0059	kg
	triethylene glycol	2.6784	kg
	water, completely softened	64.243	kg
	wire drawing, steel	1.4726	kg
	electricity, medium voltage	7.9068	kW h
	heat, district or industrial, natural gas	3.5581	MJ
output	AOX, Adsorbable Organic Halogen as Cl	0.0005	kg
	BOD5, Biological Oxygen Demand	0.0292	kg
	COD, Chemical Oxygen Demand	0.0292	kg
	Copper, ion	0.0001	kg
	DOC, Dissolved Organic Carbon	0.011	kg
	multi-Si wafer	0.9884	m2
	Nickel, ion	0.0001	kg
	Nitrogen	0.0098	kg
	Phosphate	0.0005	kg
	TOC, Total Organic Carbon	0.011	kg
	waste, from silicon wafer production	0.168	kg
	Water	0.0642	m3
mc-Si ingot productio n			
input	argon, liquid	0.3004	kg
	ceramic tile	0.3855	kg
	electricity, medium voltage	21.744	kW h
	helium	0.0001	kg
	nitrogen, liquid	0.0527	kg
	silicon, electronics grade	0.187	kg
	silicon, solar grade	1.0979	kg
	silicone factory	0	Item (s)
	Water, cooling, unspecified natural origin	5.6346	m3
output	silicon, multi-Si, casted	1.1269	kg
	Water	5.6346	m3

	Water, river	2.1785	m3
output	Acetic acid	0.0573	kg
	BOD5, Biological Oxygen Demand	0.1454	kg
	COD, Chemical Oxygen Demand	0.1454	kg
	DOC, Dissolved Organic Carbon	0.0423	kg
	Fluoride	0.0025	kg
	Hydrocarbons, unspecified	0.0243	kg
	Hydroxide	0.0079	kg
	Nitrogen	0.0097	kg
	silicon, single crystal, Czochralski process, photovoltaics	1.0623	kg
	TOC, Total Organic Carbon	0.0423	kg
	waste, from silicon wafer production, inorganic	3.8629	kg
	Water	4.7568	m3

Supplementary Methods 2: Electricity mix

We obtain time series electricity generation by source data of each region from decades ago to recent years[8]. Typical electricity sources include coal, oil, natural gas, hydropower, nuclear, wind, etc. As the annual electricity generation increases for most regions over the years, the shares of sources also vary dramatically based on each region's individual productivity advancements, energy demand requests, energy policy emphasis, and carbon neutrality target. The data in 2010 and 2015 were retrieved directly, while the data used to represent the 2020 case were the closest to 2020.

The energy structure of different countries has differed drastically in the past ten years. While China's coal demand and production capacity remained high, the government pushed to reduce emissions, improve air quality, and enhance the competitiveness of wind and solar PV in the electricity network. In Malaysia, the share of natural gas in the power mix continued to decrease by roughly 10% every five years, from 67% in 2005 to 57% in 2010, and from 47% in 2015 to 37% in 2019, responding to the policy of switching to coal and hydro fuels. Japan was one of the few countries with a decrease in power generation from 2010 to 2020 based on its energy security, economic efficiency, as well as environmental sustainability principles. Vietnam relied more on hydropower than other countries, but its coal-fired capacity was on the rise until 2019. South Korea's energy sector was characterized by a predominance of fossil fuels, including coal, natural gas, and nuclear power, on par with Taiwan's. Singapore, Thailand, and Mexico relied heavily on natural gas as their primary power source. In contrast, Germany relied on a wide variety of fuels, with a significant increase in the contribution of wind power from 2010 to 2020. While most regions in the world still rely on natural gas and coal as major sources of electricity, most have set goals to diversify the energy mix soon, phasing out traditional fossil fuels and increasing the share of renewables.

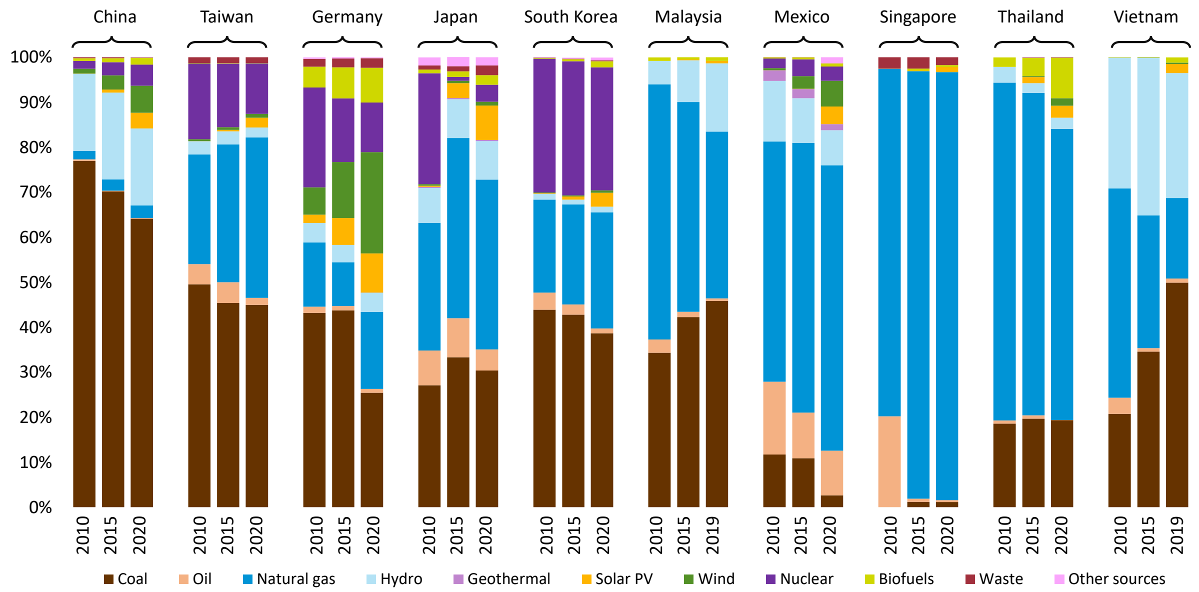

Supplementary Fig. 10 Electricity generation by energy sources among c-Si PV suppliers from 2010 to 2020. The composition of other sources differs by location. For instance, other sources in Japan represent electricity generated with procured steam, while other sources in Mexico mainly represent electricity generated from recovered waste heat from industry.

The U.S. energy policy landscape has also varied fundamentally over time in its ability to provide a renewable, affordable, and environmentally sustainable energy system to be anticipated in the future, as shown in Supplementary Fig. 11. The U.S. fuel mix for power generation has undergone a considerable shift, with coal power declining from around 20% to 13% and renewables rapidly growing from around 20% to 38%, driven by lower costs and policy support. Policies at the state and federal levels have encouraged significant investment in renewable energy generation. The fuel mix projection assumes no consideration of long-term structural changes in electricity demand accounting for the pandemic[9]. New capacity additions come primarily from natural gas and, increasingly, renewable technologies, as generating capacities of coal and nuclear retire. Incentives for renewables and declining technology costs support intense competition with natural gas. With the share of natural gas generating remaining relatively stable and the contribution of coal and nuclear halving, renewables will more than double their share from 2020 to 2050. Electricity demand will grow at a modest rate throughout the forecast period. The AEO projects that renewable generation will grow faster than overall electricity demand through 2050. Wind marks the main contributor to the growth in renewable generation before 2024, accounting for more than two-thirds of the growth over this period. After the production tax credit for wind energy were to be phased out at the end of 2024, solar power would account for almost three-quarters of the growth in renewable generation.

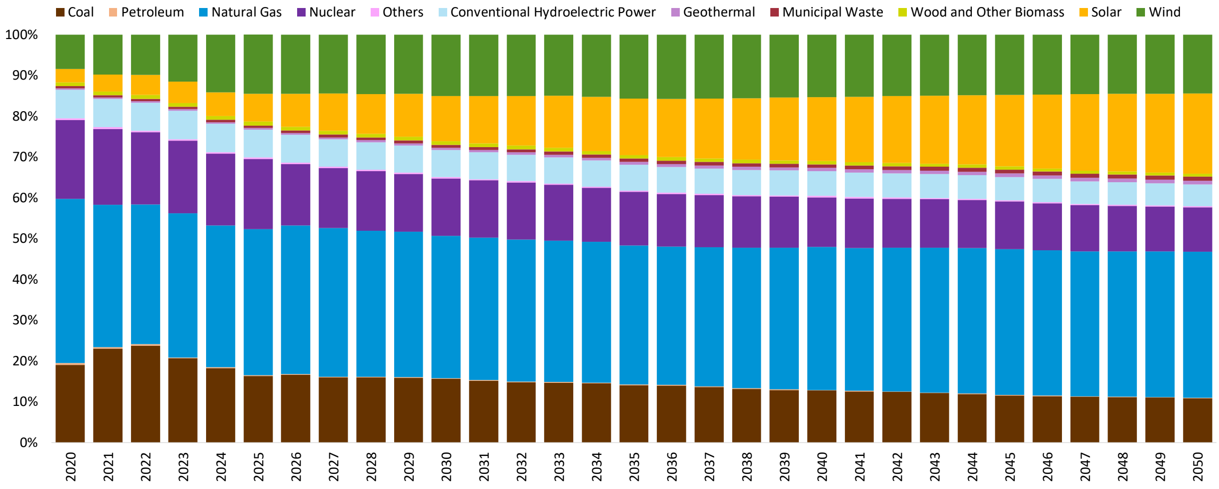

Supplementary Fig. 11 Electricity generation by energy sources data in the U.S. from 2020 to 2050. Based on projections, with the share of natural gas generating remaining relatively stable and the contribution of coal and nuclear halving, renewables is anticipated to more than double their shares from 2020 to 2050. Other sources include other renewables other than the ones listed.

Supplementary Results 4: life cycle impact assessment results

Table S2 presents the global warming potential (GWP) of all cases and scenarios with a detailed stage breakdown. The intergovernmental panel on climate change (IPCC) 2013 method is selected as one of the primary LCIA methods in this study, as it is imperative to comprehend the implications of promising energy suppliers: PVs. Several prevailing sustainability metrics are calculated based on GWP. GWP was illustrated over an integrated time horizon of 100 years, using the impact assessment method described by IPCC 2013 GWP 100a[10,11]. We first normalize the life cycle GWP on a 1 m^2 PV module basis, then analyze the corresponding life cycle stage breakdown.

Table S2. Characterization factors extracted from the Ecoinvent database. The impact category is global warming potential (GWP). The GWP stage breakdown is presented.

Year, Manufacturing location, Process	IPCC GWP 100a (kg CO_2 eq)	GWP Stage Breakdown							
		Metallurgical Grade Si	Electronics Grade Si	Solar Grade Si	Crystals/Ingots	Wafer	Cell	Panel	Transportation
2010 CN photovoltaic panel production, single-Si wafer	395.8	19.9	20.8	113.2	109.9	39.1	38.6	51.4	3.0
2010 DE photovoltaic panel production, single-Si wafer	293.2	15.0	14.3	78.7	72.2	33.0	28.1	49.7	2.0
2010 JP photovoltaic panel production, single-Si wafer	277.0	13.8	12.2	67.6	69.8	35.6	26.1	49.3	2.6
2010 MX photovoltaic panel production, single-Si wafer	290.2	14.6	13.1	72.5	75.6	36.1	27.9	49.6	0.7
2010 MY photovoltaic panel production, single-Si wafer	353.5	17.7	17.6	96.1	95.6	37.8	34.1	50.7	3.8
2010 TW photovoltaic panel production, single-Si wafer	346.7	17.3	17.3	94.5	93.0	37.6	33.3	50.5	3.1
2010 Average Global photovoltaic panel production, single-Si wafer	347.6	17.4	17.3	94.8	93.3	37.5	33.5	50.6	3.1
2015 CN photovoltaic panel production, single-Si wafer	374.2	18.8	19.2	104.8	102.6	38.4	36.3	51.0	3.0
2015 JP photovoltaic panel production, single-Si wafer	316.7	15.8	15.1	83.2	83.0	36.7	30.2	50.0	2.6
2015 KR photovoltaic panel production, single-Si wafer	307.3	15.3	14.4	79.2	80.1	36.5	29.3	49.9	2.7
2015 MX photovoltaic panel production, single-Si wafer	280.6	14.1	12.4	68.7	72.3	35.8	26.9	49.5	0.7
2015 MY photovoltaic panel production, single-Si wafer	358.5	18.0	17.9	98.1	97.3	38.0	34.7	50.8	3.8
2015 TW photovoltaic panel production, single-Si wafer	341.8	17.1	16.9	92.6	91.4	37.5	32.8	50.5	3.1
2015 Average Global photovoltaic panel production, single-Si wafer	352.5	17.7	17.6	96.2	95.3	37.8	34.1	50.7	3.1
2020 CN photovoltaic panel production, single-Si wafer	355.1	17.8	17.8	97.3	96.3	37.9	34.4	50.7	3.0
2020 KR photovoltaic panel production, single-Si wafer	298.4	14.9	13.7	75.7	77.1	36.2	28.4	49.7	2.7
2020 MY photovoltaic panel production, single-Si wafer	353.9	17.7	17.6	96.3	95.8	37.9	34.2	50.7	3.8
2020 SG photovoltaic panel production, single-Si wafer	255.2	12.6	10.5	58.6	62.1	34.9	23.7	48.9	3.8

2020 TH photovoltaic panel production, single-Si wafer	304.7	15.2	14.0	77.3	79.3	36.4	29.1	49.8	3.5
2020 VN photovoltaic panel production, single-Si wafer	297.3	14.8	13.5	74.5	76.8	36.2	28.3	49.7	3.5
2020 Average Global photovoltaic panel production, single-Si wafer	324.3	16.2	15.5	85.0	85.8	37.0	31.1	50.2	3.5
2020 US photovoltaic panel production, single-Si wafer	248.5	12.5	10.2	57.2	61.5	34.9	23.5	48.7	
2025 US photovoltaic panel production, single-Si wafer	233.2	11.7	9.1	51.2	56.4	34.4	22.0	48.4	
2030 US photovoltaic panel production, single-Si wafer	229.9	11.5	8.9	49.9	55.3	34.3	21.6	48.4	
2035 US photovoltaic panel production, single-Si wafer	224.1	11.2	8.5	47.7	53.3	34.2	21.0	48.3	
2040 US photovoltaic panel production, single-Si wafer	221.1	11.1	8.2	46.5	52.3	34.1	20.7	48.2	
2045 US photovoltaic panel production, single-Si wafer	218.1	10.9	8.0	45.3	51.3	34.0	20.4	48.2	
2050 US photovoltaic panel production, single-Si wafer	216.1	10.8	7.9	44.6	50.6	33.9	20.2	48.1	
2010 CN photovoltaic panel production, ribbon-Si	248.1	13.0	13.6	73.9		56.2	38.6	51.4	1.5
2010 DE photovoltaic panel production, ribbon-Si	185.5	9.8	9.3	51.4		36.1	28.1	49.7	1.0
2010 JP photovoltaic panel production, ribbon-Si	175.4	9.0	8.0	44.1		37.6	26.1	49.3	1.3
2010 MX photovoltaic panel production, ribbon-Si	183.6	9.6	8.6	47.3		40.3	27.9	49.6	0.3
2010 MY photovoltaic panel production, ribbon-Si	222.1	11.6	11.5	62.8		49.7	34.2	50.7	1.9
2010 TW photovoltaic panel production, ribbon-Si	218.1	11.3	11.3	61.7		48.4	33.3	50.5	1.5
2010 Average Global photovoltaic panel production, ribbon-Si	218.6	11.4	11.3	61.9		48.4	33.5	50.6	1.5
2015 CN photovoltaic panel production, ribbon-Si	234.9	12.3	12.5	68.4		52.9	36.3	51.0	1.5
2015 JP photovoltaic panel production, ribbon-Si	199.8	10.3	9.9	54.3		43.7	30.2	50.0	1.3
2015 KR photovoltaic panel production, ribbon-Si	194.0	10.0	9.4	51.7		42.4	29.3	49.9	1.3
2015 MX photovoltaic panel production, ribbon-Si	177.7	9.2	8.1	44.9		38.8	26.9	49.5	0.3
2015 MY photovoltaic panel production, ribbon-Si	225.2	11.7	11.7	64.0		50.4	34.7	50.8	1.9
2015 TW photovoltaic panel production, ribbon-Si	215.1	11.1	11.0	60.4		47.7	32.8	50.5	1.5
2015 Average Global photovoltaic panel production, ribbon-Si	221.6	11.5	11.5	62.8		49.5	34.1	50.7	1.5
2020 CN photovoltaic panel production, ribbon-Si	223.1	11.6	11.6	63.5		49.9	34.4	50.7	1.5
2020 KR photovoltaic panel production, ribbon-Si	188.5	9.7	9.0	49.4		41.0	28.4	49.7	1.3
2020 MY photovoltaic panel production, ribbon-Si	222.3	11.6	11.5	62.8		49.7	34.2	50.7	1.9
2020 SG photovoltaic panel production, ribbon-Si	161.9	8.2	6.9	38.3		34.0	23.7	48.9	1.9
2020 TH photovoltaic panel production, ribbon-Si	192.3	9.9	9.2	50.5		42.1	29.1	49.8	1.7
2020 VN photovoltaic panel production, ribbon-Si	187.7	9.7	8.8	48.6		40.9	28.3	49.7	1.7
2020 Average Global photovoltaic panel production, ribbon-Si	204.3	10.6	10.1	55.5		45.1	31.1	50.2	1.7

2020 US photovoltaic panel production, ribbon-Si	158.3	8.1	6.7	37.3		33.7	23.5	48.9	
2025 US photovoltaic panel production, ribbon-Si	149.0	7.6	6.0	33.4		31.4	22.0	48.6	
2030 US photovoltaic panel production, ribbon-Si	146.9	7.5	5.8	32.6		30.8	21.6	48.6	
2035 US photovoltaic panel production, ribbon-Si	143.4	7.3	5.5	31.1		29.9	21.0	48.5	
2040 US photovoltaic panel production, ribbon-Si	141.5	7.2	5.4	30.4		29.4	20.7	48.4	
2045 US photovoltaic panel production, ribbon-Si	139.7	7.1	5.2	29.6		29.0	20.4	48.4	
2050 US photovoltaic panel production, ribbon-Si	138.4	7.0	5.1	29.1		28.7	20.2	48.3	
2010 CN photovoltaic panel production, multi-Si wafer	328.4	22.5	23.5	128.0	22.4	39.1	38.6	51.4	3.0
2010 DE photovoltaic panel production, multi-Si wafer	249.6	17.0	16.1	89.0	14.6	33.1	28.1	49.7	2.0
2010 JP photovoltaic panel production, multi-Si wafer	232.3	15.5	13.8	76.4	12.9	35.6	26.1	49.3	2.6
2010 MX photovoltaic panel production, multi-Si wafer	242.0	16.5	14.8	81.9	14.3	36.1	27.9	49.6	0.7
2010 MY photovoltaic panel production, multi-Si wafer	294.1	20.0	19.9	108.7	19.1	37.9	34.1	50.7	3.8
2010 TW photovoltaic panel production, multi-Si wafer	289.0	19.6	19.5	106.8	18.4	37.6	33.3	50.5	3.1
2010 Average Global photovoltaic panel production, multi-Si wafer	289.7	19.7	19.6	107.2	18.6	37.5	33.5	50.6	3.1
2015 CN photovoltaic panel production, multi-Si wafer	310.8	21.2	21.7	118.4	20.7	38.5	36.3	51.0	3.0
2015 JP photovoltaic panel production, multi-Si wafer	264.7	17.8	17.1	94.1	16.0	36.8	30.2	50.0	2.6
2015 KR photovoltaic panel production, multi-Si wafer	256.8	17.3	16.3	89.5	15.3	36.5	29.3	49.9	2.7
2015 MX photovoltaic panel production, multi-Si wafer	234.1	16.0	14.0	77.7	13.5	35.8	26.9	49.5	0.7
2015 MY photovoltaic panel production, multi-Si wafer	298.1	20.3	20.3	110.8	19.5	38.0	34.7	50.8	3.8
2015 TW photovoltaic panel production, multi-Si wafer	285.0	19.3	19.1	104.7	18.0	37.5	32.8	50.5	3.1
2015 Average Global photovoltaic panel production, multi-Si wafer	293.3	20.0	19.9	108.8	19.0	37.8	34.1	50.7	3.1
2020 CN photovoltaic panel production, multi-Si wafer	295.3	20.1	20.1	110.0	19.2	37.9	34.4	50.7	3.0
2020 KR photovoltaic panel production, multi-Si wafer	249.6	16.8	15.5	85.6	14.6	36.2	28.4	49.7	2.7
2020 MY photovoltaic panel production, multi-Si wafer	294.4	20.1	19.9	108.8	19.1	37.9	34.2	50.7	3.8
2020 SG photovoltaic panel production, multi-Si wafer	214.9	14.2	11.9	66.3	11.0	34.9	23.7	48.9	3.8
2020 TH photovoltaic panel production, multi-Si wafer	254.5	17.2	15.9	87.4	15.2	36.4	29.1	49.8	3.5
2020 VN photovoltaic panel production, multi-Si wafer	248.5	16.8	15.3	84.2	14.6	36.2	28.3	49.7	3.5
2020 Average Global photovoltaic panel production, multi-Si wafer	270.4	18.3	17.5	96.1	16.7	37.0	31.1	50.2	3.5
2020 US photovoltaic panel production, multi-Si wafer	208.5	14.1	11.6	64.6	10.9	34.9	23.5	48.9	
2025 US photovoltaic panel production, multi-Si wafer	196.1	13.2	10.3	57.9	9.7	34.4	22.0	48.6	
2030 US photovoltaic panel production, multi-Si wafer	193.5	13.0	10.0	56.5	9.4	34.3	21.6	48.6	

2035 US photovoltaic panel production, multi-Si wafer	188.7	12.7	9.6	53.9	8.9	34.2	21.0	48.5
2040 US photovoltaic panel production, multi-Si wafer	186.2	12.5	9.3	52.6	8.7	34.1	20.7	48.4
2045 US photovoltaic panel production, multi-Si wafer	183.8	12.3	9.1	51.3	8.5	34.0	20.4	48.4
2050 US photovoltaic panel production, multi-Si wafer	182.2	12.2	8.9	50.4	8.3	33.9	20.2	48.3

Table S3 presents the cumulative energy demand of all cases and scenarios with a detailed energy stage breakdown. Primary energy consumption is selected as another primary LCIA method in this study, as it is imperative to comprehend the energy implications of promising energy suppliers: PVs. Several prevailing sustainability metrics are calculated based on the CED.

Table S3. Characterization factors extracted from the Ecoinvent database. The impact category is cumulative energy demand (CED). The CED stage breakdown is presented.

		Energy Stage Breakdown							
Year, Manufacturing location, Process	CED (MJ)	Metallurgical Grade Si	Electronics Grade Si	Solar Grade Si	Crystals/Ingots	Wafer	Cell	Panel	Transportation
2010 CN photovoltaic panel production, single-Si wafer	4739.3	224.2	223.6	1238.9	1232.7	686.2	396.6	696.5	40.6
2010 DE photovoltaic panel production, single-Si wafer	4880.5	231.2	243.0	1345.8	1282.6	634.0	410.8	705.4	27.7
2010 JP photovoltaic panel production, single-Si wafer	4927.6	233.5	238.9	1320.6	1292.9	691.5	415.3	699.6	35.2
2010 MX photovoltaic panel production, single-Si wafer	4574.8	218.2	212.1	1178.0	1193.5	682.8	384.4	694.5	11.2
2010 MY photovoltaic panel production, single-Si wafer	5063.7	240.6	246.2	1359.5	1338.8	695.5	429.5	702.0	51.6
2010 TW photovoltaic panel production, single-Si wafer	5092.6	241.3	251.1	1385.4	1343.5	695.9	430.9	702.3	42.2
2010 Average Global photovoltaic panel production, single-Si wafer	5000.2	237.1	243.7	1346.3	1316.5	690.1	422.5	701.2	42.9
2015 CN photovoltaic panel production, single-Si wafer	4650.3	219.6	217.0	1204.0	1203.0	683.7	387.4	695.0	40.6
2015 JP photovoltaic panel production, single-Si wafer	4606.0	216.9	215.4	1195.6	1184.9	682.1	381.8	694.0	35.2
2015 KR photovoltaic panel production, single-Si wafer	5244.6	250.2	261.3	1439.5	1401.3	700.9	448.9	705.3	37.3
2015 MX photovoltaic panel production, single-Si wafer	4535.9	216.2	209.3	1162.9	1180.4	681.7	380.4	693.8	11.2
2015 MY photovoltaic panel production, single-Si wafer	5000.5	237.3	241.6	1334.8	1317.7	693.6	422.9	700.9	51.6
2015 TW photovoltaic panel production, single-Si wafer	5010.5	237.1	245.1	1353.4	1316.0	693.5	422.4	700.8	42.2
2015 Average Global photovoltaic panel production, single-Si wafer	4833.3	228.9	230.3	1275.0	1263.3	688.9	406.1	698.1	42.6
2020 CN photovoltaic panel production, single-Si wafer	4580.5	216.1	211.8	1176.5	1179.9	681.6	380.2	693.8	40.6
2020 KR photovoltaic panel production, single-Si wafer	5127.1	244.1	252.8	1394.1	1361.6	697.4	436.6	703.2	37.3
2020 MY photovoltaic panel production, single-Si wafer	4899.3	232.1	234.2	1295.3	1283.8	690.7	412.4	699.2	51.6
2020 SG photovoltaic panel production, single-Si wafer	4405.7	205.2	200.3	1115.0	1109.1	675.5	358.2	690.1	52.3
2020 TH photovoltaic panel production, single-Si wafer	4419.9	207.4	199.5	1111.0	1123.7	676.8	362.8	690.9	47.8
2020 VN photovoltaic panel production, single-Si wafer	4238.0	198.3	185.8	1038.2	1064.5	671.6	344.4	687.8	47.4
2020 Average Global photovoltaic panel production, single-Si wafer	4654.7	219.6	216.7	1202.3	1202.5	683.6	387.2	695.0	47.9
2020 US photovoltaic panel production, single-Si wafer	4479.2	212.8	208.0	1155.9	1158.9	679.8	373.6	690.3	

2025 US photovoltaic panel production, single-Si wafer	4269.8	202.0	192.6	1074.4	1088.6	673.7	351.8	686.6	
2030 US photovoltaic panel production, single-Si wafer	4119.6	194.2	181.7	1016.1	1038.1	669.3	336.2	684.0	
2035 US photovoltaic panel production, single-Si wafer	4024.6	189.3	174.7	979.4	1006.1	666.5	326.2	682.4	
2040 US photovoltaic panel production, single-Si wafer	3964.0	186.2	170.3	955.9	985.6	664.7	319.9	681.3	
2045 US photovoltaic panel production, single-Si wafer	3911.1	183.4	166.5	935.4	967.8	663.2	314.4	680.4	
2050 US photovoltaic panel production, single-Si wafer	3854.8	180.5	162.3	913.4	949.0	661.5	308.5	679.4	
2010 CN photovoltaic panel production, ribbon-Si	2885.7	146.4	146.0	808.8		671.4	396.6	696.5	20.1
2010 DE photovoltaic panel production, ribbon-Si	3003.6	150.9	158.7	878.6		685.5	410.8	705.4	13.7
2010 JP photovoltaic panel production, ribbon-Si	3002.4	152.4	156.0	862.1		699.5	415.3	699.6	17.4
2010 MX photovoltaic panel production, ribbon-Si	2787.5	142.4	138.5	769.0		653.1	384.4	694.5	5.5
2010 MY photovoltaic panel production, ribbon-Si	3083.2	157.0	160.8	887.5		720.9	429.5	702.0	25.5
2010 TW photovoltaic panel production, ribbon-Si	3103.0	157.5	164.0	904.4		723.1	431.0	702.3	20.8
2010 Average Global photovoltaic panel production, ribbon-Si	3047.6	154.8	159.1	878.9		709.9	422.5	701.2	21.2
2015 CN photovoltaic panel production, ribbon-Si	2831.1	143.4	141.7	786.0		657.5	387.4	695.0	20.1
2015 JP photovoltaic panel production, ribbon-Si	2805.1	141.6	140.7	780.5		649.1	381.8	694.0	17.4
2015 KR photovoltaic panel production, ribbon-Si	3196.3	163.3	170.6	939.7		750.0	448.9	705.3	18.4
2015 MX photovoltaic panel production, ribbon-Si	2763.7	141.1	136.6	759.2		647.0	380.4	693.8	5.5
2015 MY photovoltaic panel production, ribbon-Si	3044.4	154.9	157.7	871.4		711.0	422.9	700.9	25.5
2015 TW photovoltaic panel production, ribbon-Si	3052.7	154.8	160.0	883.5		710.3	422.4	700.8	20.8
2015 Average Global photovoltaic panel production, ribbon-Si	2943.1	149.5	150.4	832.3		685.7	406.1	698.1	21.0
2020 CN photovoltaic panel production, ribbon-Si	2788.2	141.1	138.3	768.0		646.8	380.2	693.8	20.1
2020 KR photovoltaic panel production, ribbon-Si	3124.2	159.3	165.0	910.1		731.5	436.6	703.2	18.4
2020 MY photovoltaic panel production, ribbon-Si	2982.4	151.5	152.9	845.6		695.2	412.4	699.2	25.5
2020 SG photovoltaic panel production, ribbon-Si	2680.6	133.9	130.8	727.9		613.8	358.3	690.1	25.8
2020 TH photovoltaic panel production, ribbon-Si	2688.8	135.4	130.3	725.3		620.6	362.8	690.9	23.6
2020 VN photovoltaic panel production, ribbon-Si	2577.2	129.5	121.3	677.7		593.0	344.4	687.8	23.4
2020 Average Global photovoltaic panel production, ribbon-Si	2832.9	143.3	141.5	784.9		657.3	387.2	695.0	23.7
2020 US photovoltaic panel production, ribbon-Si	2732.6	138.9	135.8	754.6		637.0	373.6	692.7	
2025 US photovoltaic panel production, ribbon-Si	2604.2	131.9	125.8	701.4		604.2	351.8	689.1	
2030 US photovoltaic panel production, ribbon-Si	2512.0	126.8	118.6	663.3		580.7	336.2	686.4	
2035 US photovoltaic panel production, ribbon-Si	2453.8	123.6	114.1	639.4		565.7	326.2	684.8	

2040 US photovoltaic panel production, ribbon-Si	2416.6	121.5	111.2	624.0		556.2	319.9	683.7	
2045 US photovoltaic panel production, ribbon-Si	2384.2	119.7	108.7	610.6		547.9	314.4	682.8	
2050 US photovoltaic panel production, ribbon-Si	2349.6	117.8	106.0	596.3		539.1	308.5	681.8	
2010 CN photovoltaic panel production, multi-Si wafer	3964.0	253.5	252.7	1400.6	237.1	686.4	396.6	696.5	40.6
2010 DE photovoltaic panel production, multi-Si wafer	4083.4	261.4	274.8	1521.4	247.9	634.2	410.8	705.4	27.7
2010 JP photovoltaic panel production, multi-Si wafer	4120.4	264.0	270.1	1492.9	251.5	691.7	415.3	699.6	35.2
2010 MX photovoltaic panel production, multi-Si wafer	3819.1	246.6	239.8	1331.7	227.8	683.0	384.4	694.5	11.2
2010 MY photovoltaic panel production, multi-Si wafer	4228.5	272.0	278.4	1536.9	262.5	695.7	429.5	702.0	51.6
2010 TW photovoltaic panel production, multi-Si wafer	4257.9	272.8	283.9	1566.2	263.6	696.1	430.9	702.3	42.2
2010 Average Global photovoltaic panel production, multi-Si wafer	4179.4	268.0	275.5	1521.9	257.1	690.3	422.5	701.2	42.9
2015 CN photovoltaic panel production, multi-Si wafer	3891.6	248.3	245.3	1361.1	230.0	683.9	387.4	695.0	40.6
2015 JP photovoltaic panel production, multi-Si wafer	3859.4	245.2	243.5	1351.6	225.7	682.3	381.8	694.0	35.2
2015 KR photovoltaic panel production, multi-Si wafer	4375.6	282.8	295.4	1627.4	277.4	701.1	448.8	705.3	37.3
2015 MX photovoltaic panel production, multi-Si wafer	3787.5	244.4	236.6	1314.6	224.6	681.9	380.4	693.8	11.2
2015 MY photovoltaic panel production, multi-Si wafer	4177.1	268.3	273.1	1508.9	257.4	693.8	422.9	700.9	51.6
2015 TW photovoltaic panel production, multi-Si wafer	4191.2	268.0	277.1	1529.9	257.1	693.7	422.4	700.8	42.2
2015 Average Global photovoltaic panel production, multi-Si wafer	4040.9	258.8	260.4	1441.3	244.4	689.1	406.1	698.1	42.6
2020 CN photovoltaic panel production, multi-Si wafer	3834.6	244.3	239.5	1329.9	224.5	681.8	380.2	693.8	40.6
2020 KR photovoltaic panel production, multi-Si wafer	4280.4	275.9	285.7	1576.0	268.0	697.7	436.6	703.2	37.3
2020 MY photovoltaic panel production, multi-Si wafer	4094.9	262.4	264.7	1464.3	249.3	690.9	412.4	699.2	51.6
2020 SG photovoltaic panel production, multi-Si wafer	3702.7	231.9	226.4	1260.5	207.6	675.7	358.2	690.1	52.3
2020 TH photovoltaic panel production, multi-Si wafer	3705.5	234.5	225.5	1256.0	211.1	677.0	362.8	690.9	47.8
2020 VN photovoltaic panel production, multi-Si wafer	3556.2	224.2	210.1	1173.6	196.9	671.8	344.4	687.8	47.4
2020 Average Global photovoltaic panel production, multi-Si wafer	3896.1	248.2	244.9	1359.1	229.9	683.8	387.2	695.0	47.9
2020 US photovoltaic panel production, multi-Si wafer	3748.2	240.6	235.1	1306.7	219.5	680.0	373.6	692.7	
2025 US photovoltaic panel production, multi-Si wafer	3578.2	228.4	217.8	1214.6	202.7	673.9	351.8	689.1	
2030 US photovoltaic panel production, multi-Si wafer	3456.3	219.6	205.4	1148.7	190.6	669.5	336.2	686.4	
2035 US photovoltaic panel production, multi-Si wafer	3379.4	214.0	197.6	1107.2	182.9	666.7	326.2	684.8	
2040 US photovoltaic panel production, multi-Si wafer	3330.2	210.4	192.6	1080.7	178.1	664.9	319.9	683.7	
2045 US photovoltaic panel production, multi-Si wafer	3287.3	207.3	188.2	1057.5	173.8	663.4	314.4	682.8	
2050 US photovoltaic panel production, multi-Si wafer	3241.6	204.1	183.5	1032.6	169.3	661.7	308.5	681.8	

Table S4 presents the cumulative energy demand (CED) with a detailed impact analysis. Types of energy consumption include non-renewable biomass, renewable water, non-renewable nuclear, renewable biomass, and other renewables such as wind, solar, geothermal, etc. Most energy consumptions come from fossil energy, while the amount of non-renewable biomass is negligible, 1/40000 as much as the energy impact from fossil energy.

Table S4. Characterization factors extracted from the Ecoinvent database. The impact category is cumulative energy demand (CED). The CED impact analysis is presented.

Year, Manufacturing location, Process	CED (MJ)	Energy Impact Analysis					
		Biomass(non-renewable)	Fossil(non-renewable)	Water(renewable)	Nuclear(non-renewable)	Biomass(renewable)	Others (wind, solar, geothermal)(renewable)
2010 CN photovoltaic panel production, single-Si wafer	4739.3	0.1	4107.0	260.0	213.8	128.2	30.3
2010 DE photovoltaic panel production, single-Si wafer	4880.5	0.0	3585.5	109.4	989.1	96.2	100.1
2010 JP photovoltaic panel production, single-Si wafer	4927.6	0.1	3533.8	152.4	1097.1	116.3	27.9
2010 MX photovoltaic panel production, single-Si wafer	4574.8	0.1	3966.0	219.9	234.2	80.7	73.8
2010 MY photovoltaic panel production, single-Si wafer	5063.7	0.1	4652.0	130.3	145.9	116.9	18.5
2010 TW photovoltaic panel production, single-Si wafer	5092.6	0.1	4148.9	92.8	748.1	79.1	23.6
2010 Average Global photovoltaic panel production, single-Si wafer	5000.2	0.1	4219.1	135.1	516.4	100.7	29.0
2015 CN photovoltaic panel production, single-Si wafer	4650.3	0.1	3920.9	282.0	255.4	139.3	52.6
2015 JP photovoltaic panel production, single-Si wafer	4606.0	0.1	4063.2	161.7	214.4	136.0	30.5
2015 KR photovoltaic panel production, single-Si wafer	5244.6	0.1	3840.4	77.1	1212.9	92.5	21.7
2015 MX photovoltaic panel production, single-Si wafer	4535.9	0.1	3880.8	182.4	292.5	88.2	91.8
2015 MY photovoltaic panel production, single-Si wafer	5000.5	0.1	4557.4	172.8	145.8	105.8	18.6
2015 TW photovoltaic panel production, single-Si wafer	5010.5	0.1	4161.5	92.4	652.9	78.2	25.4
2015 Average Global photovoltaic panel production, single-Si wafer	4833.3	0.1	4180.6	200.2	299.6	116.0	36.8
2020 CN photovoltaic panel production, single-Si wafer	4580.5	0.1	3756.7	259.3	319.4	162.1	83.0
2020 KR photovoltaic panel production, single-Si wafer	5127.1	0.1	3774.9	77.6	1128.8	120.1	25.6
2020 MY photovoltaic panel production, single-Si wafer	4899.3	0.1	4380.3	234.0	145.6	120.7	18.6
2020 SG photovoltaic panel production, single-Si wafer	4405.7	0.1	4100.5	70.0	143.8	73.2	18.1
2020 TH photovoltaic panel production, single-Si wafer	4419.9	0.1	4059.9	100.3	148.5	74.6	36.6
2020 VN photovoltaic panel production, single-Si wafer	4238.0	0.1	3602.8	373.4	143.7	96.6	21.5
2020 Average Global photovoltaic panel production, single-Si wafer	4654.7	0.1	4044.0	222.5	252.7	108.3	27.2

2020 US photovoltaic panel production, single-Si wafer	4479.2	0.1	3230.1	146.6	882.2	101.8	118.5
2025 US photovoltaic panel production, single-Si wafer	4269.8	0.1	3038.1	144.8	800.8	100.6	185.5
2030 US photovoltaic panel production, single-Si wafer	4119.6	0.1	2999.7	142.5	684.0	99.4	193.9
2035 US photovoltaic panel production, single-Si wafer	4024.6	0.1	2935.7	139.6	646.8	98.7	203.7
2040 US photovoltaic panel production, single-Si wafer	3964.0	0.1	2914.9	136.5	613.1	97.1	202.3
2045 US photovoltaic panel production, single-Si wafer	3911.1	0.1	2892.8	132.7	591.9	95.8	197.7
2050 US photovoltaic panel production, single-Si wafer	3854.8	0.1	2874.0	129.1	562.1	94.8	194.7
2010 CN photovoltaic panel production, ribbon-Si	2885.7	0.0	2491.3	163.2	125.7	87.6	17.7
2010 DE photovoltaic panel production, ribbon-Si	3003.6	0.0	2190.1	71.4	611.9	68.5	61.7
2010 JP photovoltaic panel production, ribbon-Si	3002.4	0.0	2140.8	97.2	667.7	80.4	16.3
2010 MX photovoltaic panel production, ribbon-Si	2787.5	0.0	2407.8	138.6	138.2	58.5	44.5
2010 MY photovoltaic panel production, ribbon-Si	3083.2	0.0	2824.3	83.6	84.0	80.7	10.5
2010 TW photovoltaic panel production, ribbon-Si	3103.0	0.0	2517.6	60.6	453.6	57.5	13.7
2010 Average Global photovoltaic panel production, ribbon-Si	3047.6	0.0	2561.1	86.6	312.0	70.8	17.0
2015 CN photovoltaic panel production, ribbon-Si	2831.1	0.0	2377.2	176.7	151.2	94.5	31.5
2015 JP photovoltaic panel production, ribbon-Si	2805.1	0.0	2465.7	103.0	126.1	92.4	17.9
2015 KR photovoltaic panel production, ribbon-Si	3196.3	0.0	2328.4	51.0	738.7	65.7	12.5
2015 MX photovoltaic panel production, ribbon-Si	2763.7	0.0	2355.5	115.6	174.0	63.1	55.5
2015 MY photovoltaic panel production, ribbon-Si	3044.4	0.0	2766.3	109.7	84.0	73.9	10.6
2015 TW photovoltaic panel production, ribbon-Si	3052.7	0.0	2525.3	60.4	395.2	57.0	14.8
2015 Average Global photovoltaic panel production, ribbon-Si	2943.1	0.0	2536.3	126.5	178.4	80.1	21.7
2020 CN photovoltaic panel production, ribbon-Si	2788.2	0.0	2276.4	162.8	190.5	108.4	50.1
2020 KR photovoltaic panel production, ribbon-Si	3124.2	0.0	2288.2	51.3	687.1	82.7	14.8
2020 MY photovoltaic panel production, ribbon-Si	2982.4	0.0	2657.7	147.2	83.9	83.0	10.6
2020 SG photovoltaic panel production, ribbon-Si	2680.6	0.0	2487.0	46.7	82.8	53.8	10.3
2020 TH photovoltaic panel production, ribbon-Si	2688.8	0.0	2461.6	65.2	85.6	54.7	21.6
2020 VN photovoltaic panel production, ribbon-Si	2577.2	0.0	2181.1	232.8	82.7	68.2	12.3
2020 Average Global photovoltaic panel production, ribbon-Si	2832.9	0.0	2451.8	140.2	149.6	75.4	15.9
2020 US photovoltaic panel production, ribbon-Si	2732.6	0.0	1960.2	93.8	535.3	71.4	71.8
2025 US photovoltaic panel production, ribbon-Si	2604.2	0.0	1842.4	92.7	485.4	70.7	112.9
2030 US photovoltaic panel production, ribbon-Si	2512.0	0.0	1818.9	91.3	413.7	70.0	118.1

2035 US photovoltaic panel production, ribbon-Si	2453.8	0.0	1779.7	89.6	390.9	69.5	124.1
2040 US photovoltaic panel production, ribbon-Si	2416.6	0.0	1766.9	87.6	370.2	68.5	123.3
2045 US photovoltaic panel production, ribbon-Si	2384.2	0.0	1753.4	85.3	357.2	67.7	120.5
2050 US photovoltaic panel production, ribbon-Si	2349.6	0.0	1741.9	83.1	338.9	67.1	118.6
2010 CN photovoltaic panel production, multi-Si wafer	3964.0	0.1	3449.0	209.9	165.3	116.6	23.1
2010 DE photovoltaic panel production, multi-Si wafer	4083.4	0.0	3066.6	87.5	763.0	89.8	76.4
2010 JP photovoltaic panel production, multi-Si wafer	4120.4	0.1	2985.0	122.5	884.6	107.1	21.2
2010 MX photovoltaic panel production, multi-Si wafer	3819.1	0.0	3324.0	176.9	181.7	78.0	58.4
2010 MY photovoltaic panel production, multi-Si wafer	4228.5	0.1	3892.9	104.4	110.1	107.4	13.6
2010 TW photovoltaic panel production, multi-Si wafer	4257.9	0.1	3488.6	74.0	600.8	76.8	17.7
2010 Average Global photovoltaic panel production, multi-Si wafer	4179.4	0.1	3545.1	108.4	409.8	94.2	21.8
2015 CN photovoltaic panel production, multi-Si wafer	3891.6	0.1	3297.7	227.7	199.1	125.7	41.3
2015 JP photovoltaic panel production, multi-Si wafer	3859.4	0.1	3416.8	130.2	165.9	123.1	23.3
2015 KR photovoltaic panel production, multi-Si wafer	4375.6	0.1	3232.8	61.2	977.7	87.6	16.2
2015 MX photovoltaic panel production, multi-Si wafer	3787.5	0.0	3254.9	146.6	228.9	84.1	73.0
2015 MY photovoltaic panel production, multi-Si wafer	4177.1	0.1	3816.0	138.9	110.0	98.4	13.6
2015 TW photovoltaic panel production, multi-Si wafer	4191.2	0.1	3499.0	73.7	523.2	76.0	19.2
2015 Average Global photovoltaic panel production, multi-Si wafer	4040.9	0.1	3509.2	161.3	235.2	106.7	28.4
2020 CN photovoltaic panel production, multi-Si wafer	3834.6	0.1	3164.1	209.2	251.1	144.2	66.0
2020 KR photovoltaic panel production, multi-Si wafer	4280.4	0.1	3179.8	61.6	909.5	110.1	19.3
2020 MY photovoltaic panel production, multi-Si wafer	4094.9	0.1	3672.2	188.7	109.9	110.5	13.6
2020 SG photovoltaic panel production, multi-Si wafer	3702.7	0.0	3453.6	55.5	108.5	71.9	13.2
2020 TH photovoltaic panel production, multi-Si wafer	3705.5	0.0	3411.9	80.0	112.2	73.1	28.3
2020 VN photovoltaic panel production, multi-Si wafer	3556.2	0.0	3039.2	301.8	108.3	90.9	16.0
2020 Average Global photovoltaic panel production, multi-Si wafer	3896.1	0.1	3398.8	179.3	197.0	100.5	20.6
2020 US photovoltaic panel production, multi-Si wafer	3748.2	0.0	2732.1	117.9	708.2	95.1	94.8
2025 US photovoltaic panel production, multi-Si wafer	3578.2	0.0	2576.1	116.4	642.1	94.1	149.3
2030 US photovoltaic panel production, multi-Si wafer	3456.3	0.0	2545.1	114.6	547.1	93.2	156.2
2035 US photovoltaic panel production, multi-Si wafer	3379.4	0.0	2493.2	112.3	517.0	92.6	164.2
2040 US photovoltaic panel production, multi-Si wafer	3330.2	0.0	2476.5	109.7	489.6	91.3	163.1
2045 US photovoltaic panel production, multi-Si wafer	3287.3	0.0	2458.6	106.7	472.4	90.3	159.4

2050 US photovoltaic panel production, multi-Si wafer	3241.6	0.0	2443.3	103.7	448.1	89.5	156.9

Table S5 presents the original EPBT and carbon emission factor estimated results without normalization of all outsourced cases and reshored scenarios. EPBT, the time needed to generate as much energy as consumed during the production stages, is an essential metric adopted widely in characterizing the energy sustainability of PV technologies. EPBT is dominated by energy embedded in raw materials and energy consumed in manufacturing products. EROI, the amount of energy expended to produce a certain amount of energy, is another critical metric proportional to the inverse of EPBT. Besides metrics that describe the energy use, the carbon emission factor or life cycle carbon emission factor, the total amount of GHG emission mainly induced from material production and PV manufacturing is also a crucial metric describing the climate change impact of the PV system. When calculating these metrics, we account for the geographical and temporal influence on input parameters, including solar irradiation, module efficiency, etc.

Table S5. Original EPBT and carbon emission factor results (without normalization)

Case / Scenario	Technology	EPBT（years)	EROI	Carbon Emission Factor (kg CO2 per kWh)
2010 Outsourced Case (China)	sc-Si	2.07	14.5	69.3
2010 Outsourced Case (Germany)	sc-Si	2.14	14.0	51.3
2010 Outsourced Case (Japan)	sc-Si	2.16	13.9	48.5
2010 Outsourced Case (Mexico)	sc-Si	2.00	15.0	50.8
2010 Outsourced Case (Malaysia)	sc-Si	2.22	13.5	61.9
2010 Outsourced Case (Taiwan)	sc-Si	2.23	13.5	60.7
2010 Outsourced Case	sc-Si	2.19	13.7	60.9
2015 Outsourced Case (China)	sc-Si	1.68	17.9	53.9
2015 Outsourced Case (Japan)	sc-Si	1.66	18.1	45.7
2015 Outsourced Case (South Korea)	sc-Si	1.89	15.9	44.3
2015 Outsourced Case (Mexico)	sc-Si	1.63	18.3	40.4
2015 Outsourced Case (Malaysia)	sc-Si	1.80	16.6	51.7
2015 Outsourced Case (Taiwan)	sc-Si	1.81	16.6	49.3
2015 Outsourced Case	sc-Si	1.74	17.2	50.8
2020 Outsourced Case (China)	sc-Si	1.37	21.9	42.5
2020 Outsourced Case (South Korea)	sc-Si	1.53	19.6	35.7
2020 Outsourced Case (Malaysia)	sc-Si	1.46	20.5	42.3
2020 Outsourced Case (Singapore)	sc-Si	1.32	22.8	30.5
2020 Outsourced Case (Thailand)	sc-Si	1.32	22.7	36.4
2020 Outsourced Case (Vietnam)	sc-Si	1.27	23.7	35.5
2020 Outsourced Case	sc-Si	1.39	21.6	38.8
2020 Reshored Scenario	sc-Si	1.34	22.4	29.7
2025 Reshored Scenario	sc-Si	1.28	23.5	27.9
2030 Reshored Scenario	sc-Si	1.23	24.4	27.5
2035 Reshored Scenario	sc-Si	1.20	24.9	26.8
2040 Reshored Scenario	sc-Si	1.18	25.3	26.4

2045 Reshored Scenario	sc-Si	1.17	25.7	26.1
2050 Reshored Scenario	sc-Si	1.15	26.0	25.8
2010 Outsourced Case (China)	r-Si	1.61	18.7	55.3
2010 Outsourced Case (Germany)	r-Si	1.67	17.9	41.3
2010 Outsourced Case (Japan)	r-Si	1.67	17.9	39.1
2010 Outsourced Case (Mexico)	r-Si	1.55	19.3	40.9
2010 Outsourced Case (Malaysia)	r-Si	1.72	17.5	49.5
2010 Outsourced Case (Taiwan)	r-Si	1.73	17.4	48.6
2010 Outsourced Case	r-Si	1.70	17.7	48.7
2015 Outsourced Case (China)	r-Si	1.33	22.5	44.3
2015 Outsourced Case (Japan)	r-Si	1.32	22.7	37.7
2015 Outsourced Case (South Korea)	r-Si	1.51	19.9	36.6
2015 Outsourced Case (Mexico)	r-Si	1.30	23.0	33.5
2015 Outsourced Case (Malaysia)	r-Si	1.43	20.9	42.5
2015 Outsourced Case (Taiwan)	r-Si	1.44	20.8	40.6
2015 Outsourced Case	r-Si	1.39	21.6	41.8
2020 Outsourced Case (China)	r-Si	1.14	26.3	36.5
2020 Outsourced Case (South Korea)	r-Si	1.28	23.5	30.8
2020 Outsourced Case (Malaysia)	r-Si	1.22	24.6	36.3
2020 Outsourced Case (Singapore)	r-Si	1.10	27.4	26.5
2020 Outsourced Case (Thailand)	r-Si	1.10	27.3	31.4
2020 Outsourced Case (Vietnam)	r-Si	1.05	28.5	30.7
2020 Outsourced Case	r-Si	1.16	25.9	33.4
2020 Reshored Scenario	r-Si	1.12	26.9	25.9
2025 Reshored Scenario	r-Si	1.06	28.2	24.3
2030 Reshored Scenario	r-Si	1.03	29.2	24.0
2035 Reshored Scenario	r-Si	1.00	29.9	23.4
2040 Reshored Scenario	r-Si	0.99	30.4	23.1
2045 Reshored Scenario	r-Si	0.97	30.8	22.8
2050 Reshored Scenario	r-Si	0.96	31.3	22.6
2010 Outsourced Case (China)	mc-Si	1.87	16.1	61.9
2010 Outsourced Case (Germany)	mc-Si	1.92	15.6	47.1
2010 Outsourced Case (Japan)	mc-Si	1.94	15.4	43.8
2010 Outsourced Case (Mexico)	mc-Si	1.80	16.7	45.6
2010 Outsourced Case (Malaysia)	mc-Si	1.99	15.1	55.4
2010 Outsourced Case (Taiwan)	mc-Si	2.01	14.9	54.5
2010 Outsourced Case	mc-Si	1.97	15.2	54.6
2015 Outsourced Case (China)	mc-Si	1.49	20.1	47.6
2015 Outsourced Case (Japan)	mc-Si	1.48	20.3	40.5
2015 Outsourced Case (South Korea)	mc-Si	1.68	17.9	39.3
2015 Outsourced Case (Mexico)	mc-Si	1.45	20.7	35.9
2015 Outsourced Case (Malaysia)	mc-Si	1.60	18.8	45.7
2015 Outsourced Case (Taiwan)	mc-Si	1.61	18.7	43.7

2015 Outsourced Case	mc-Si	1.55	19.4	44.9
2020 Outsourced Case (China)	mc-Si	1.31	23.0	40.2
2020 Outsourced Case (South Korea)	mc-Si	1.46	20.6	34.0
2020 Outsourced Case (Malaysia)	mc-Si	1.39	21.5	40.1
2020 Outsourced Case (Singapore)	mc-Si	1.26	23.8	29.3
2020 Outsourced Case (Thailand)	mc-Si	1.26	23.8	34.6
2020 Outsourced Case (Vietnam)	mc-Si	1.21	24.8	33.8
2020 Outsourced Case	mc-Si	1.33	22.6	36.8
2020 Reshored Scenario	mc-Si	1.28	23.5	28.4
2025 Reshored Scenario	mc-Si	1.22	24.6	26.7
2030 Reshored Scenario	mc-Si	1.18	25.5	26.3
2035 Reshored Scenario	mc-Si	1.15	26.1	25.7
2040 Reshored Scenario	mc-Si	1.13	26.5	25.4
2045 Reshored Scenario	mc-Si	1.12	26.8	25.0
2050 Reshored Scenario	mc-Si	1.10	27.2	24.8

Supplementary References

1. International Trade Commission *Crystalline Silicon Photovoltaic Cells, Whether or Not Partially or Fully Assembled Into Other Products* (2021). (Accessed 10/30/2021).
2. Wang, M. et al. Breaking down barriers on PV trade will facilitate global carbon mitigation. *Nature Communications* **12,** 6820 (2021).
3. Goldthau, A. & Hughes, L. Protect global supply chains for low-carbon technologies. *Nature* **585,** 28-30 (2020).
4. Fthenakis, V. & Leccisi, E. Updated sustainability status of crystalline silicon‐based photovoltaic systems: Life‐cycle energy and environmental impact reduction trends. *Progress in Photovoltaics: Research and Applications* **29,** 1068-1077 (2021).
5. Fthenakis, V., Kim, H. C., Held, M., Raugei, M. & Krones, J. Update of PV energy payback times and life-cycle greenhouse gas emissions. *24th European Photovoltaic Solar Energy Conference and Exhibition* 21-25 (2009).
6. Rabaia, M. K. H. et al. Environmental impacts of solar energy systems: A review. *Science of The Total Environment* **754,** 141989 (2021).
7. Fu, Y., Liu, X. & Yuan, Z. Life-cycle assessment of multi-crystalline photovoltaic (PV) systems in China. *Journal of Cleaner Production* **86,** 180-190 (2015).
8. World Energy Outlook 2020. (International Energy Agency, 2020).
9. Annual Energy Outlook 2021. (U.S. Energy Information Administration, U.S. Department of Energy, 2021).
10. Forster, P. et al. *Changes in atmospheric constituents and in radiative forcing*. Vol. **39** (2007).
11. Pearce, W., Holmberg, K., Hellsten, I. & Nerlich, B. Climate change on Twitter: Topics, communities and conversations about the 2013 IPCC Working Group 1 report. *PloS one* **9,** e94785 (2014).
